# Tissue tropism and mRNA expression profiles of selected innate immunity-related genes following experimental tick-borne encephalitis virus and louping ill virus infection of sheep

**DOI:** 10.1128/jvi.00651-26

**Published:** 2026-06-25

**Authors:** Nadjah Radia Adjadj, Mara Rocchi, Laurent Mostin, Nick De Regge

**Affiliations:** 1 Unit of Viral Reemerging Enzootic and Bee Diseases, Sciensano 54513 , Brussels, Belgium; 2 Moredun Research Institute 6485, Penicuik, United Kingdom; 3 Experimental Center Machelen, Sciensano, Machelen, Belgium; 4 Unit of Exotic Viruses and Vector-borne Diseases, Sciensano 54513 , Brussels, Belgium; University of North Carolina at Chapel Hill, Chapel Hill, North Carolina, USA

**Keywords:** tick-borne encephalitis virus, louping ill virus, sheep, *in vivo*, cytokine, immune response

## Abstract

Tick-borne encephalitis virus (TBEV) and louping ill virus (LIV) are genetically and antigenically closely related tick-borne flaviviruses responsible for different disease outcomes in sheep. In order to determine whether key components of the innate immunity could contribute to the observed difference in virus dissemination and disease outcome, 8-month-old sheep were inoculated intradermally with TBEV Neudoerfl strain or LIV LI/31 strain. Only LIV-infected sheep developed fever and clinical signs. No TBEV RNA was detected in serum and examined tissues, except for low amounts in the skin, prescapular lymph nodes, and spleen. In contrast, high LIV RNA load and infectious virus were found in serum and various tissues, indicating a tropism for lymphoid organs and the brain. The highest LIV viral loads were found in the brain stem, the cerebellum, and the thalamus. The analysis of mRNA expression profiles of selected cytokines and chemokines showed a weak to moderate innate antiviral immune response in the prescapular lymph nodes of TBEV-infected sheep. Conversely, a strong upregulation of several interferon-stimulated genes, chemokines, and pro-inflammatory cytokines was induced in the prescapular lymph nodes, tonsils, medulla oblongata, cerebellum, and thalamus of LIV-infected animals. Neutralizing antibodies were detected in both LIV- and TBEV-infected sheep. These data suggest that the innate and humoral immune responses were sufficient to block TBEV replication early after infection but insufficient to stop LIV entry and replication in the brain, as supported by the high viral loads at euthanasia, and likely contribute to the induced clinical symptoms.

**IMPORTANCE:**

Although LIV and TBEV are closely related tick-borne flaviviruses, the outcome of an infection in sheep is considerably different. Here, we show that the effectiveness of the innate immune response to limit virus replication corresponds to a specific clinical outcome. TBEV infection seemed to be efficiently controlled at the level of the prescapular lymph node by a moderate interferon-related cytokine response. This early control prevented likely further TBEV spread and entry in the CNS. In contrast, LIV was capable of replicating to high viral loads in prescapular lymph nodes, tonsils, and brain tissues despite the strong innate immune responses induced in these tissues, which probably contributed to the observed clinical signs. This further suggests that LIV has adapted to better circumvent innate immune responses than TBEV and that the clinical manifestations can be attributed to a dysregulated response.

## INTRODUCTION

Tick-borne encephalitis virus (TBEV) and louping ill virus (LIV) are tick-borne neurotropic viruses within the tick-borne encephalitis serocomplex, belonging to the genus *Orthoflavivirus* (family *Flaviviridae*), which was formerly classified within the genus *Flavivirus*. The latter includes mosquito-borne neurotropic viruses, such as West Nile virus (WNV), Japanese encephalitis virus (JEV), yellow fever virus (YFV), and dengue virus (DENV) (1–3). TBEV distribution spans a large area of Europe and Asia and is expanding to previously unaffected countries, whereas LIV is predominantly endemic in the United Kingdom and is focally present in other areas of Europe (such as Spain, Norway, Greece, and Turkey) (4–7). At least five subtypes of TBEV have been documented, namely European (TBEV-Eu), Far Eastern (TBEV-FE), Siberian (TBEV-Sib), Himalayan (TBEV-Him), and the recently detected Baikalian (TBEV-Bkl) (8–10). LIV, whose name derived from the Scottish word describing the “leaping gait” developed in affected animals, is divided into five subtypes: British (LIV-Brit), Irish (LIV-Ir), Spanish (LIV-Spain), Turkish sheep encephalitis virus subtype (TSEV), and Greek goat encephalitis virus subtype (GGEV) (7, 11, 12). TBEV and LIV are mainly transmitted by *Ixodes ricinus* ticks in Europe, but milk-borne infection through the consumption of unpasteurized milk and milk products originating from viremic ruminants is also possible, especially for TBEV (4, 13–15). TBEV shares 95% of amino acid identity with LIV. Despite being genetically and antigenically closely related, TBEV and LIV diverge in certain features, including their reservoir hosts and their disease outcome in humans and sheep (16–19).

Small mammals, particularly rodents, are the main reservoir hosts for TBEV, but other mammalian species, including wild boar, deer, goat, cattle, and sheep, can be infected. However, given the low levels of viremia in these species, they mainly contribute to the circulation of the virus by enabling tick survival and reproduction. TBEV is also zoonotic, but humans are considered dead-end hosts and do not play a role in virus transmission (8, 20–22). Two-thirds of TBEV human infections are asymptomatic, but in symptomatic cases, the virus can cause encephalitis with serious sequelae (23–26). Unlike humans and, more commonly, dogs, only sporadic TBEV cases have been reported in domestic and wild animals, suggesting that the immune responses in these animals could prevent the development of clinical signs. TBEV infection in sheep is generally asymptomatic and rarely causes neurological symptoms (17, 27).

LIV has been detected in goats, dogs, pigs, horses, deer, llamas, alpacas, grouse, and ptarmigans; however, the reservoir hosts are sheep, red grouse, and mountain hares. Sheep and red grouse can develop high viremia sufficient for vector-host-vector transmission and are regarded as essential for the persistence of the virus. Hares do not develop high viremia enough to infect ticks directly but have been implicated in LIV transmission through a co-feeding mechanism (28). Humans are accidental hosts of LIV, and their infection is rare and associated with professional exposure (7, 29–32). LIV infection in humans is not easily distinguishable from tick-borne encephalitis (TBE). The infection is mostly asymptomatic or results in flu-like symptoms, followed in some cases by more severe neurological manifestations (7, 20).

In sheep, weaned lambs and yearlings appear to be more susceptible to LIV infection. As for many flaviviruses, LIV induces a biphasic pyrexia pattern. The first stage is associated with viremia, and the second with neuroinvasion. The presence and absence of the virus in blood are generally correlated with the initial rise and decline in temperature, respectively (7, 33). When it occurs, neuroinvasion is characterized by depression, panting, and nibbling, and progresses to more characteristic disease signs, including muscle tremors, incoordination, circling, and ataxia. Affected animals lose their appetite and become recumbent. They ultimately develop paralysis and succumb to death. Morbidity and mortality rates vary between 5% and 60% depending on age, breed, previous exposure status, and concomitant infections, such as tick-borne fever. Survivors of the encephalitic stage may exhibit signs of torticollis and paraplegia (7).

Very few studies addressing the pathogenesis and related immune responses after LIV and TBEV infection of sheep have been conducted so far. These include early work by Doherty and Reid in the 1970s, mainly focusing on the histopathological changes and the humoral immune response related to LIV infection in sheep. More recently, Mansfield et al. (17) investigated some markers of the innate and adaptive immune responses to TBEV and LIV following subcutaneous infection in sheep (17, 34–38). They also investigated potential cross-protection between TBEV and LIV by studying whether prior exposure to either TBEV or LIV modified the outcome of infection. It was shown that exposure to TBEV did not prevent LIV infection but reduced disease severity and viremia (17). Here, we performed an explorative and descriptive *in vivo* study of tissue tropism and selected immune markers over time upon TBEV and LIV intradermal infection of sheep. This study aimed to provide, following an inoculation route mimicking the natural infection by ticks, first indications of the time course of viral entry and replication in different organs (including the brain). It also aimed to gain more insights into the innate and humoral immune response that might contribute to the clearance of the virus from the different organs over time.

## RESULTS

### Descriptive pathogenesis

#### Rectal temperature and clinical symptoms

**Fig 1:**
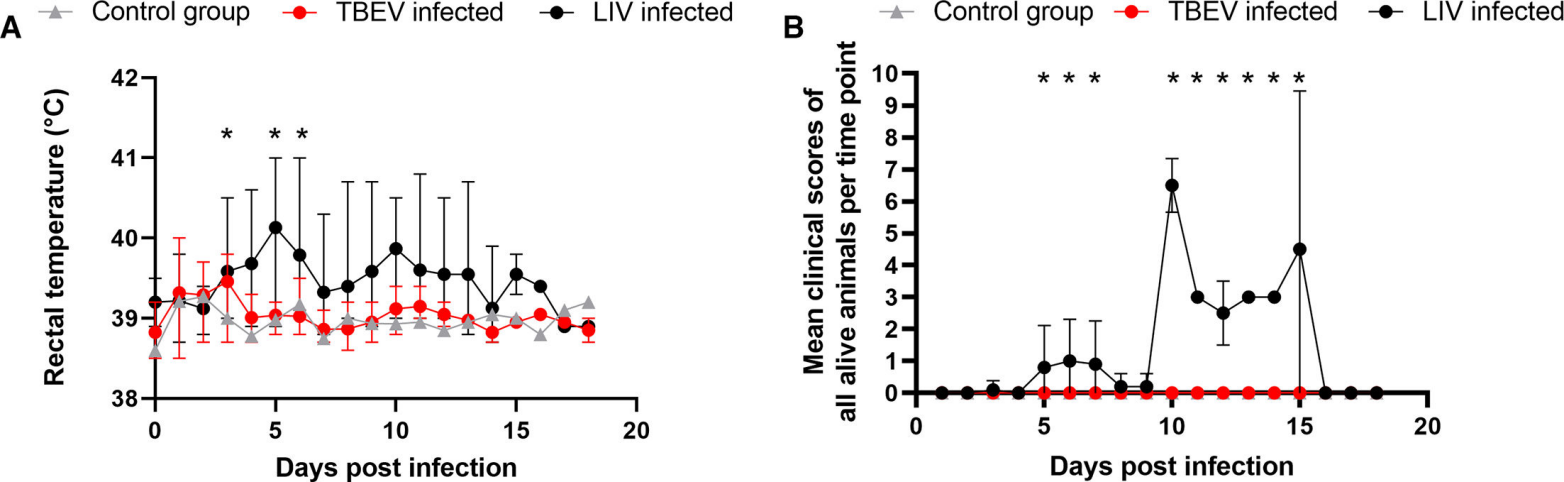
Mean rectal temperature (**A**) and mean clinical scores (**B**) of 8-month-old sheep inoculated intradermally with TBEV Neudoerfl strain (red dots) or LIV LI/31 strain (black dots) at a dose of 10^5^ TCID_50_/sheep. Mean rectal temperature and clinical scores of the control group (gray triangles) are also shown in panels A and B. The parameters were recorded daily until the end of the experiment (18 dpi). Data in panels A and B are presented as mean ± standard error (error bars). Asterisks indicate statistically significant higher temperatures (A) and clinical scores (B) compared to baseline (0 dpi in panel A; 1 dpi in panel B) within the LIV-infected group (black dots). Clinical scores in panel B were assessed according to the criteria detailed in Table S1. *N* = 42 (control group = 10, TBEV-infected = 16, LIV-infected = 16).

Control sheep and TBEV-infected sheep exhibited neither fever nor clinical symptoms over the course of the study (from 1 to 18 days post-infection [dpi]) (Fig. 1A and B). In contrast, a biphasic fever was observed in sheep infected with LIV (Fig. 1A). The first fever peak was reached at 5 dpi, with significantly higher temperatures at 3, 5, and 6 dpi compared to day 0 (Mann-Whitney U tests, *P* < 0.05). A second peak could be observed at 10 dpi, although the temperature at this peak was not significantly higher than the temperature measured at day 0 (Mann-Whitney U test, *P* = 0.122). At 5 and 7 dpi, LIV-infected sheep started showing increased respiratory rate (4/10 animals and 2/8 animals, respectively), slight depression (2/10 and 1/8, respectively), reduced appetite (1/10 and 3/8, respectively), and slight head trembling (1/10 and 1/8, respectively). At 10 dpi, more pronounced clinical manifestations were observed. These included suppressed appetite (6/6), abdominal breathing (6/6), and lethargy (5/6). At 11, 13, and 14 dpi, animals presented increased respiration rate (4/4), slight depression (4/4), and reduced appetite (4/4). At 15 dpi, one LIV-infected animal showed labored breathing, was louping and unable to stand, and was therefore euthanized earlier than planned (at 15 dpi instead of at 18 dpi). The total clinical scores of LIV-infected sheep were significantly higher at 5, 6, 7, and 10 to 15 dpi when compared to 1 dpi (Mann-Whitney U tests, *P* < 0.05). A detailed scoring card of the clinical signs is provided in Table S1.

#### TBEV and LIV detection in the skin and prescapular lymph nodes

**Fig 2:**
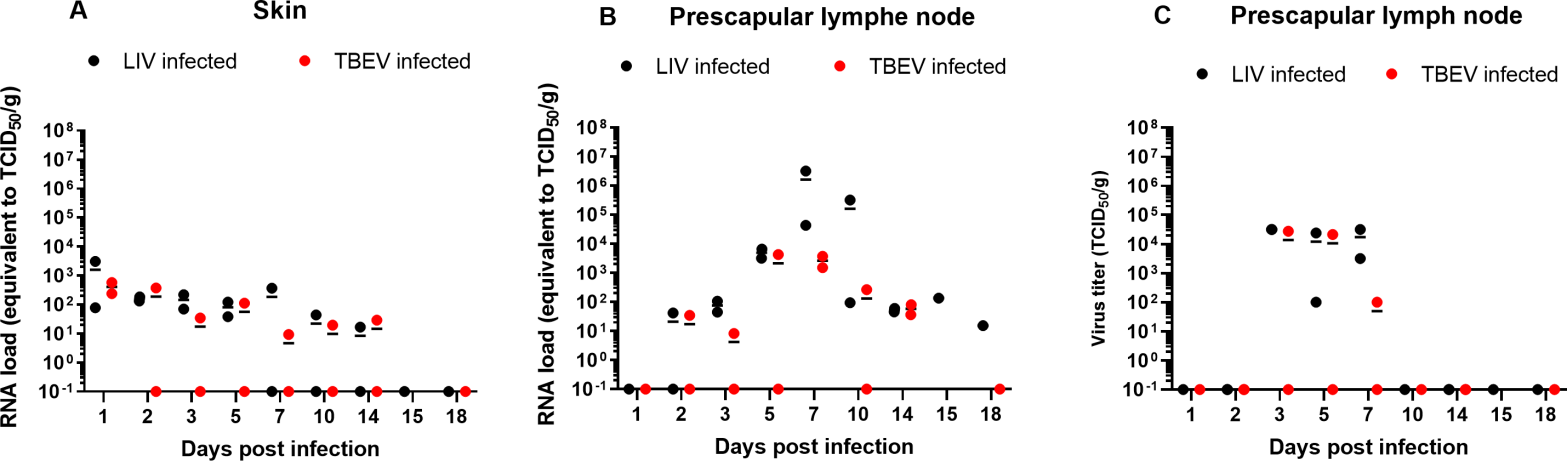
Detection of TBEV and LIV RNA by RT-qPCR in the skin (**A**) and prescapular lymph nodes (**B**) of 8-month-old sheep inoculated intradermally with TBEV Neudoerfl strain (red dots) or LIV LI/31 strain (black dots) at a dose of 10^5^ TCID_50_/sheep. Ct values were converted using a standard curve generated by testing a 10-fold serial dilution of TBEV and LIV second passage stocks with known titers and expressed as RNA load equivalent to TCID_50_/g. (**C**) Amount of infectious TBEV and LIV in the prescapular lymph nodes determined by virus titration and expressed as TCID_50_/g. Two TBEV-infected animals were euthanized at 1, 3, 5, 7, 10, 14, and 18 dpi. Two LIV-infected animals were euthanized at 1, 3, 5, 7, 10, and 14 dpi. One LIV-infected animal was euthanized earlier than planned (on 15 instead of day 18 dpi) due to reaching the humane endpoint, and one LIV-infected animal was euthanized at 18 dpi. Each dot represents one animal, and the horizontal line represents the mean.

Following intradermal infection of sheep with TBEV or LIV, viral RNA of both viruses was detected by RT-qPCR in skin biopsies collected at the initial site of inoculation from 1 dpi to 14 dpi in at least one out of the two examined animals per time point. The detected viral loads in the skin ranged between equivalent doses of 10 to 10^3^ TCID_50_/g and remained stable over time (Fig. 2A). Viral RNA of TBEV and LIV was also detected by RT-qPCR at 2 dpi in the draining (prescapular) lymph nodes of one out of the two examined sheep. TBEV RNA was detected from 2 to 14 dpi in one out of the two examined animals per time point except for 7 and 14 dpi where the prescapular lymph nodes of both examined sheep were positive (Fig. 2B). TBEV viral loads increased also from 2 to 7 dpi, although to a lesser extent compared to LIV, and then decreased from 7 dpi until the end of the experiment. From 3 dpi until the end of the experiment (18 dpi), the prescapular lymph nodes of all LIV-infected sheep were positive. An increase of LIV viral loads was observed from 2 (dose equivalent to 10^2^ TCID_50_/g) to 7 (dose equivalent to 10^6^ TCID_50_/g) dpi, followed by a decrease from 7 dpi onwards (Fig. 2B). Infectious TBEV and LIV could be isolated from some RT-qPCR-positive prescapular lymph node samples between 3 and 7 dpi. Viral titers ranged between 10^2^ and 10^4^ TCID_50_/g (Fig. 2C). Control animals were negative at all time points.

#### TBEV and LIV detection in blood, lymphoid, and visceral organs, and seroconversion

**Fig 3:**
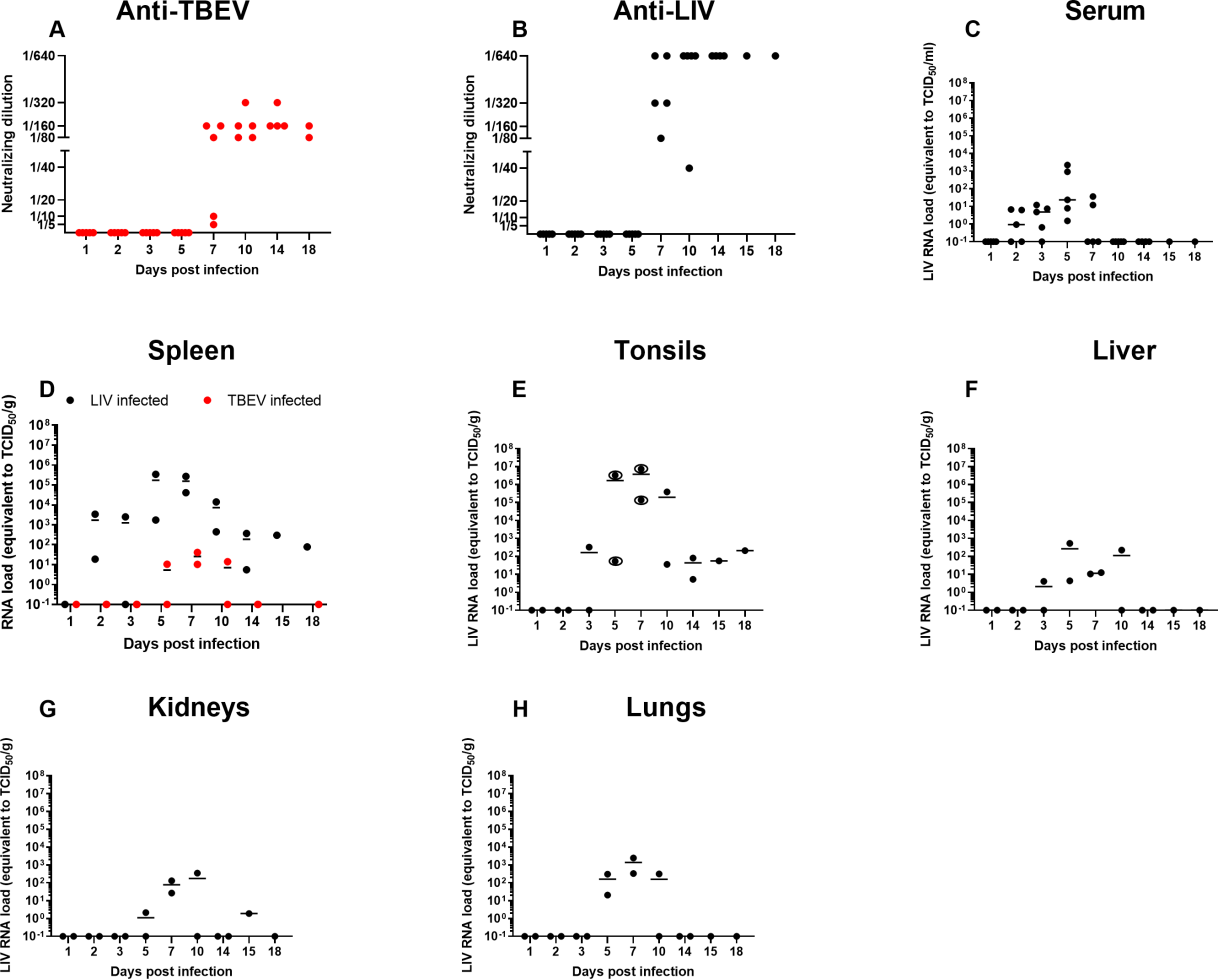
TBEV neutralizing antibodies titers measured by PRNT in serum of 8-month-old sheep infected intradermally with TBEV Neudoerfl strain at a dose of 10^5^ TCID_50_/sheep (**A**). LIV neutralizing antibodies titers measured by PRNT in serum of 8-month-old sheep infected intradermally with LIV LI/31 strain at a dose of 10^5^ TCID_50_/sheep (**B**). LIV RNA loads in serum of LIV-infected sheep measured by RT-qPCR (**C**). TBEV and LIV RNA loads in the spleen of TBEV-infected (red dots) and LIV-infected sheep (black dots) measured by RT-qPCR (**D**). LIV RNA loads in the tonsils (**E**), liver (**F**), kidneys (**G**), and lungs (**H**) of LIV-infected sheep measured by RT-qPCR. A standard curve was generated by testing a 10-fold serial dilution of TBEV and LIV second passage stocks with known titer in RT-qPCR and was used to convert Ct values in equivalent viral loads as TCID_50_/mL or TCID_50_/g. Two TBEV-infected animals were euthanized at 1, 3, 5, 7, 10, 14, and 18 dpi. Two LIV-infected animals were euthanized at 1, 3, 5, 7, 10, and 14 dpi. One LIV-infected animal was euthanized earlier than planned (on 15 instead of day 18 dpi) due to reaching the humane endpoint, and one LIV-infected animal was euthanized at 18 dpi. Each dot represents one animal, and the horizontal line represents the mean. Dots surrounded by an open circle indicate isolation-positive samples in the tonsils.

No TBEV RNA was detected in the serum of TBEV-infected sheep at any time point by RT-qPCR, although PRNT results indicated the seroconversion of sheep at 7 dpi (Fig. 3A). Conversely, LIV RNA was detected already at 2 dpi in the serum of LIV-infected sheep, and peak viremia was reached at 5 dpi (Fig. 3C). LIV RNA loads in serum dropped at 7 dpi, coinciding with the first detection of neutralizing antibodies (Fig. 3B). Antibody titers in TBEV- and LIV-infected animals reached 1/320 and 1/640, respectively (Fig. 3A and B). While both TBEV and LIV RNA were found in the spleen, LIV RNA was detected earlier (at 2 dpi as opposed to 5 dpi) and at higher viral loads (up to loads equivalent to 10^5^ TCID_50_/g in contrast to loads equivalent to 10^2^ TCID_50_/g) (Fig. 3D). TBEV RNA was detected only in the tonsils of one animal (at 7 dpi). LIV RNA, on the other hand, was detected from 3 dpi until the end of the experiment in the tonsils of 11 out of 12 animals (Fig. 3E).

The amounts of LIV RNA in the tonsils increased between 3 and 7 dpi (reaching doses more than the equivalent of 10^6^ TCID_50_/g), and LIV was isolated from tonsils at 5 and 7 dpi with titers higher than 10^4^ TCID_50_/g (Fig. 3E, see Fig. S1 for the viral titers). Unlike TBEV, which remained undetected in the visceral organs of infected animals, LIV disseminated to the liver by 3 dpi (Fig. 3F) and to the kidneys and lungs by 5 dpi (Fig. 3G and H, respectively), as measured by RT-qPCR.

#### LIV spread into the central nervous system (CNS)

**Fig 4:**
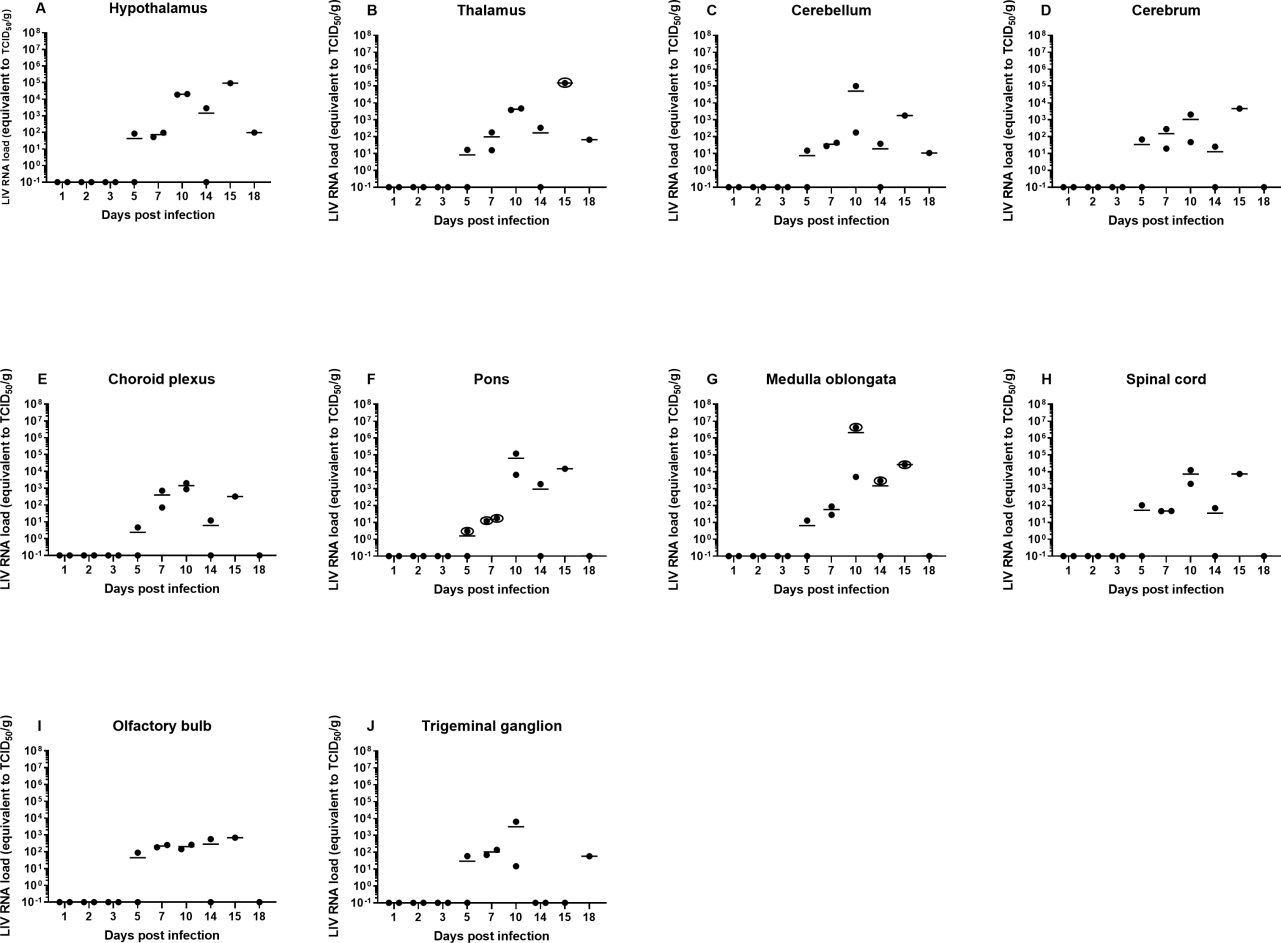
LIV distribution into the CNS. LIV RNA loads detected by RT-qPCR in the hypothalamus (**A**), thalamus (**B**), cerebellum (**C**), cerebrum (**D**), choroid plexus (**E**), pons (**F**), medulla oblongata (**G**), spinal cord (**H**), olfactory bulb (**I**), and trigeminal ganglion (**J**) of 8-month-old sheep inoculated intradermally with LIV LI/31 strain at a dose of 10^5^ TCID_50_/sheep. Ct values were converted using a standard curve generated by testing a 10-fold serial dilution of TBEV and LIV second passage stocks with known titers and expressed as RNA load equivalent to TCID_50_/g. Two LIV-infected animals were euthanized at 1, 3, 5, 7, 10, 14, and 18 dpi, with the exception of one animal euthanized earlier than planned (on 15 instead of day 18 dpi) due to reaching the humane endpoint. Each dot represents one animal, and the horizontal line represents the mean. Dots surrounded by an open circle indicate isolation-positive samples in the thalamus, pons, and medulla oblongata.

While no TBEV RNA was detected by RT-qPCR in the brain, LIV RNA was found in all examined CNS areas (hypothalamus, thalamus, cerebellum, cerebrum, choroid plexus, pons, medulla oblongata, spinal cord, olfactory bulb, and trigeminal ganglion) at 5 dpi, coinciding with peak viremia. Figure 4 shows that although viral loads differed between the brain areas, they reached their peak at 10 dpi, except in the cerebrum, the hypothalamus, and thalamus. The highest viral loads of LIV at 10 dpi were found in the medulla oblongata (load more than equivalent to 10^6^ TCID_50_/g) and the pons (load up to the equivalent of 10^5^ TCID_50_/g), followed by the cerebellum (load almost equivalent to 10^5^ TCID_50_/g). In the hypothalamus and thalamus, the highest viral loads were detected in the animal euthanized earlier than planned (at 15 dpi) due to neurological signs. In addition, the other CNS areas of this animal showed the presence of high LIV viral loads. LIV RNA loads in the olfactory bulb were stable over time and ranged between doses equivalent to 10^2^ and 10^3^ TCID_50_/g (Fig. 4). LIV isolation from RT-qPCR-positive brain tissues was successful only in some instances from the thalamus, medulla oblongata, and pons, with titers ranging between 10^2^ and 10^4^ TCID_50_/g (Fig. 4, see Fig. S1 for the viral titers).

### Pattern recognition receptors, cytokine, and chemokine mRNA expression

mRNA expression profiles of different selected cytokine categories (pattern recognition receptors [PRRs], interferons, interferon-related, and inflammasome-related cytokines, chemokines, pro-inflammatory, and anti-inflammatory cytokines) were analyzed in prescapular lymph nodes, tonsils, cerebellum, thalamus, and medulla oblongata. These organs were selected based on the results of RT-qPCR, virus isolation, and titration, in addition to their relevance in the pathogenesis of TBEV and LIV.

### mRNA expression in the prescapular lymph nodes of TBEV-infected sheep

**Fig 5:**
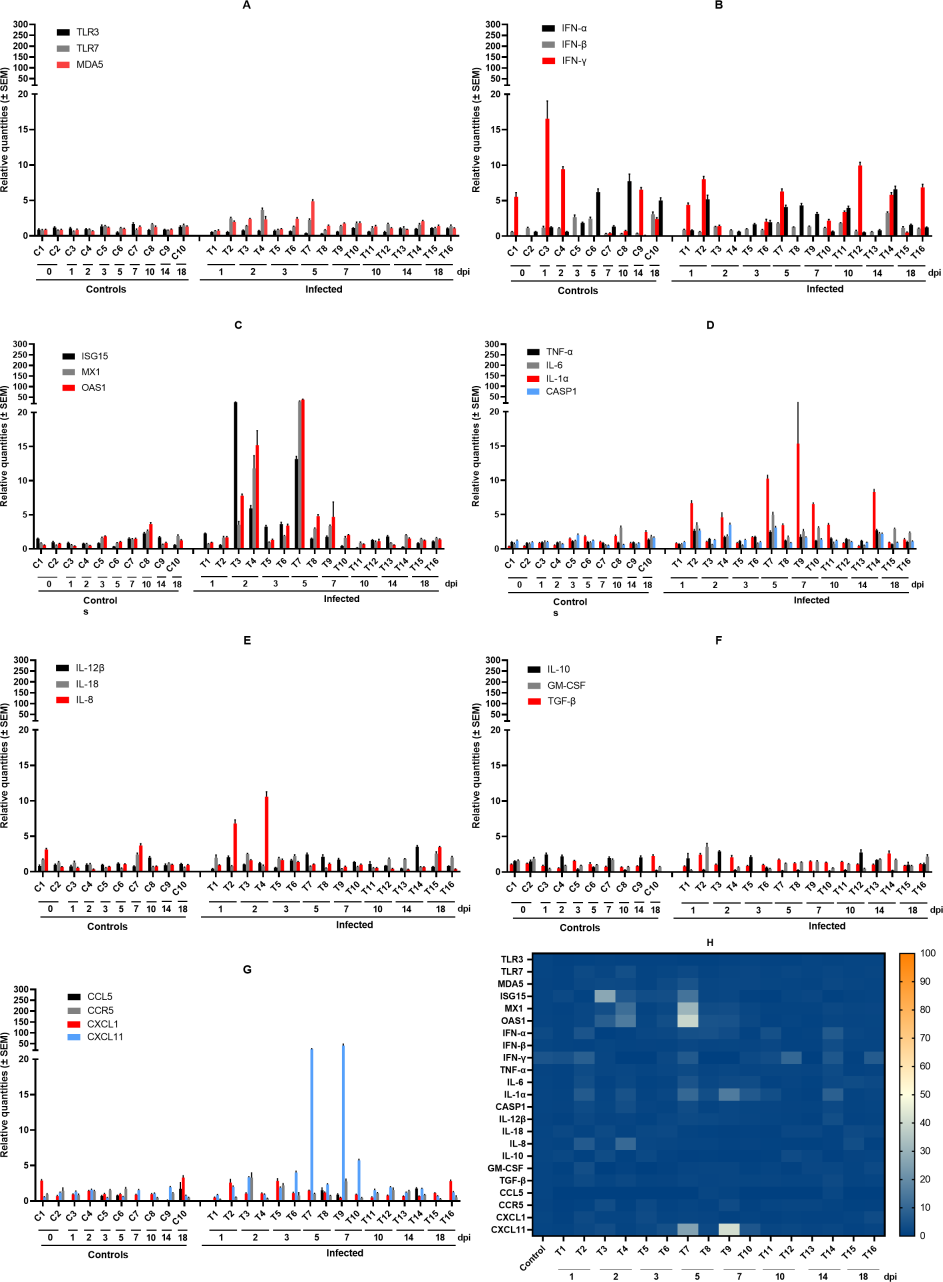
mRNA expression profiles of the selected genes in the prescapular lymph nodes of 8-month-old sheep inoculated intradermally with TBEV Neudoerfl strain at a dose of 10^5^ TCID_50_/sheep. Fold changes in mRNA expression of (**A**) TLR3, TLR7, MDA5; (**B**) IFN-α, IFN-β, IFN-γ; (**C**) ISG15, MX1, OAS1; (**D**) TNF-α, IL-6, IL-1α, CASP1; (**E**) IL-12β, IL-18, IL-8; (**F**) IL-10, GM-CSF, TGF-β; and (**G**) CCL5, CCR5, CXCL1, and CXCL11 were determined by RT-qPCR and relative quantification and are depicted over time (at 1, 2, 3, 5, 7, 10, 14, and 18 dpi). (**H**) Heat map visualization of mRNA expression changes in the selected genes. Target gene expression levels of infected animals were expressed relative to the average of the control group. Error bars represent technical replicates.

Except for a variable increase (5- to 40-fold) in the expression profiles of ISG15, MX1, OAS1, IL-1α, IL-8, and CXCL11 in some sheep at some specific time points, predominantly at 5 and 7 dpi, no clear changes in cytokine expression profiles were observed in the prescapular lymph nodes of TBEV-infected sheep (Fig. 5). The 5th and 7th dpi corresponded to the time points at which TBEV viral loads in the prescapular lymph nodes were highest (Fig. 2B).

### mRNA expression in the prescapular lymph nodes of LIV-infected sheep

**Fig 6:**
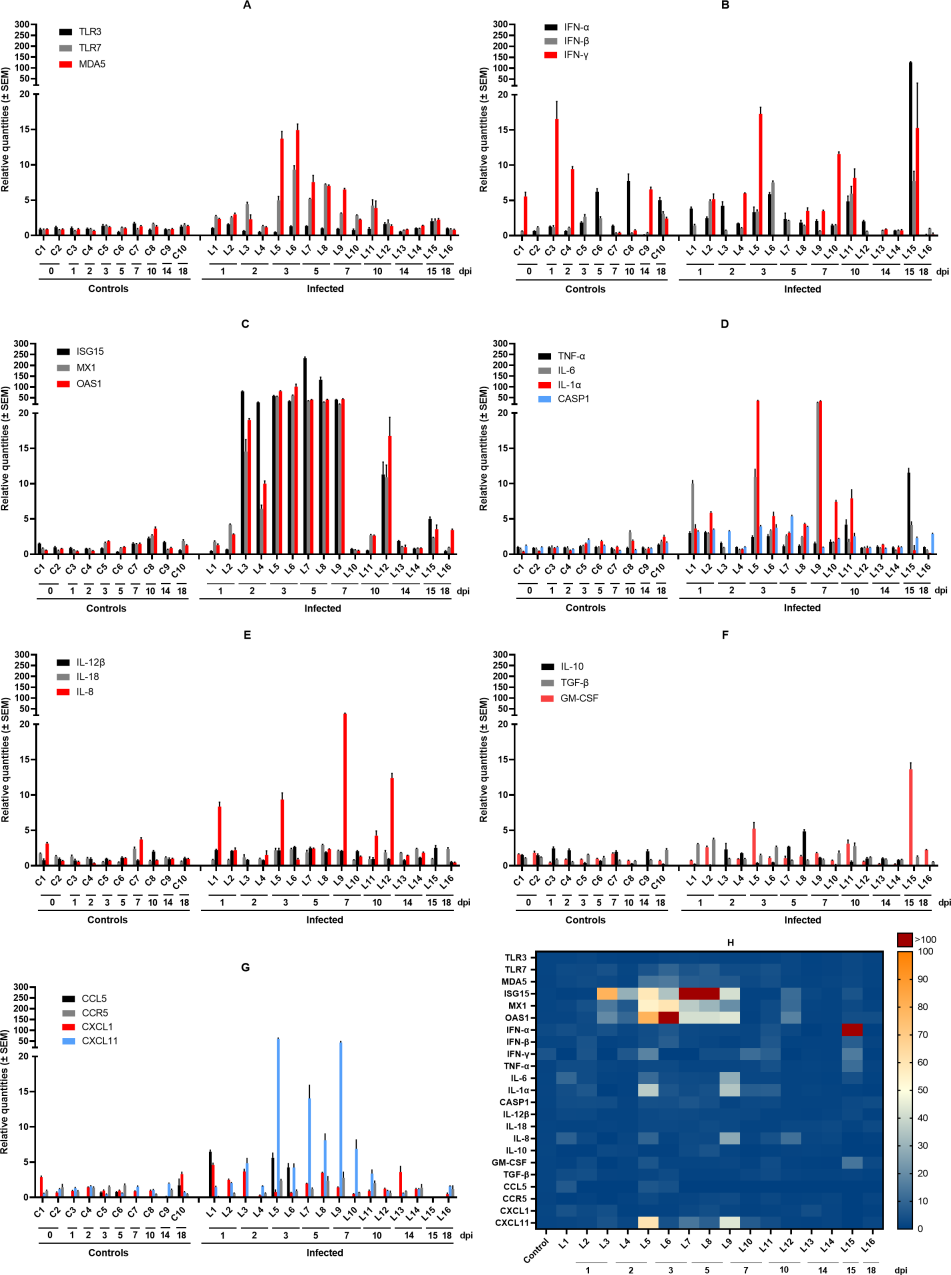
mRNA expression profiles of the selected genes in the prescapular lymph nodes of 8-month-old sheep inoculated intradermally with LIV LI/31 strain at a dose of 10^5^ TCID_50_/sheep. Fold changes in mRNA expression of (**A**) TLR3, TLR7, MDA5; (**B**) IFN-α, IFN-β, IFN-γ; (**C**) ISG15, MX1, OAS1; (**D**) TNF-α, IL-6, IL-1α, CASP1; (**E**) IL-12β, IL-18, IL-8; (**F**) IL-10, GM-CSF, TGF-β; and (**G**) CCL5, CCR5, CXCL1, and CXCL11 were determined by RT-qPCR and relative quantification and are depicted over time (at 1, 2, 3, 5, 7, 10, 14, 15, and 18 dpi). (**H**) Heat map visualization of mRNA expression changes in the selected genes. Target gene expression levels of infected animals were expressed relative to the average of the control group. Error bars represent technical replicates.

Among the genes analyzed in the prescapular lymph nodes, a consistent and strong upregulation of interferon-stimulated genes (ISGs) mRNA was observed upon LIV infection (Fig. 6C and H). ISG15 increase ranged from 30- to 80-fold between 2 and 3 dpi, with a further increase (exceeding 100- to 200-fold change) at 5 dpi. Subsequently, a 40-fold and 10-fold rise were detected at 7 and 10 dpi, respectively, in one out of the two LIV-infected sheep. From 14 dpi onwards until 18 dpi, ISG15 expression profile returned to normal (Fig. 6C). The expression profiles of MX1 and OAS1 followed the same pattern as ISG15, with OAS1 exhibiting higher levels than MX1 (Fig. 6C). Also, a low to moderate 5- to 10-fold and 7- to 15-fold increase of TLR7 and MDA5 mRNA expression, respectively, was observed between 3 dpi and 5 dpi (Fig. 6A).

No marked changes in the expression profiles of interferons (IFN-α, IFN-β, and IFN-γ) were observed except in animal L15 at 15 dpi, which had a strong rise in IFN-α mRNA expression (>120-fold) (Fig. 6B). This animal exhibited clinical signs (including no appetite, gasping, head shaking, and louping) and was euthanized earlier than foreseen (at 15 dpi instead of at 18 dpi). Moreover, the prescapular lymph node of this animal was the only tissue in which an increase in the expression of TNF-α and GM-CSF was also observed, although to a moderate extent (around 10-fold) (Fig. 6D and F). Among the other pro-inflammatory and anti-inflammatory cytokines, only changes in the expression levels of IL-6, IL-1α, and IL-8 were observed in some animals at isolated time points. The increase in the expression of IL-8 and IL-1α at 7 and 10 dpi was especially seen in animals that manifested fever and clinical signs (such as reduced or no appetite, depression, lethargy, slight head tremor, and circling). Finally, no increase in chemokine expression was observed in the prescapular lymph nodes upon LIV infection, with the exception of CXCL11, which reached more than 40-fold increase at 3 and 7 dpi in one out of the two infected animals (Fig. 6G).

### mRNA and protein expression in the tonsils of LIV-infected sheep

**Fig 7:**
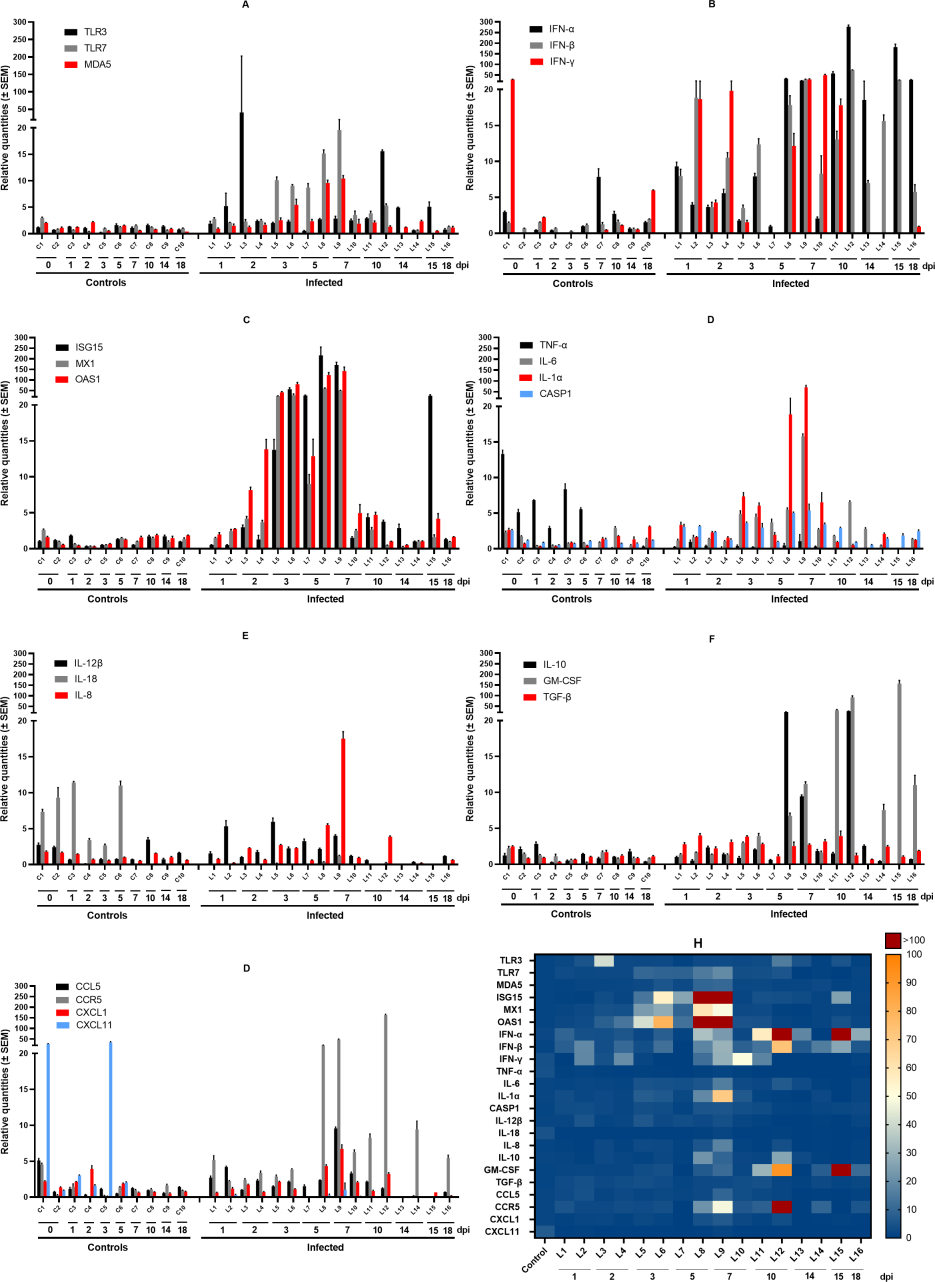
mRNA expression profiles of the selected genes in the soft palate tonsil of 8-month-old sheep inoculated intradermally with LIV LI/31 strain at a dose of 10^5^ TCID_50_/sheep. Fold changes in mRNA expression of (**A**) TLR3, TLR7, MDA5; (**B**) IFN-α, IFN-β, IFN-γ; (**C**) ISG15, MX1, OAS1; (**D**) TNF-α, IL-6, IL-1α, CASP1; (**E**) IL-12β, IL-18, IL-8; (**F**) IL-10, GM-CSF, TGF-β; and (**G**) CCL5, CCR5, CXCL1, and CXCL11 were determined by RT-qPCR, and relative quantification is depicted over time (at 1, 2, 3, 5, 7, 10, 14, 15, and 18 dpi). (**H**) Heat map visualization of mRNA expression changes in the selected genes. Target gene expression levels of infected animals were expressed relative to the average of the control group. Error bars represent technical replicates.

As described above, tonsils were among the organs in which the highest viral LIV loads were detected upon infection. This resulted in an extensive induction of the expression levels of multiple selected cytokine genes. A moderate increase in mRNA expression levels of TLR7 (8- to 20-fold change) was detected in infected animals from 3 to 7 dpi (Fig. 7A and H). Moreover, the levels of mRNA expression of IFN-α and IFN-β increased between 5 and 15 dpi, particularly in two animals, L12 and L15. In these animals, respectively, IFN-α showed a strong increase of approximately 270-fold and 180-fold, while IFN-β exhibited an increase of approximately 70-fold and 26-fold, respectively (Fig. 7B). The most consistent and strong upregulation was observed for ISGs (ISG15, Mx1, and OAS1). These were strongly upregulated in infected sheep from 3 to 7 dpi (Fig. 7C), especially in those animals with the highest LIV viral loads in the tonsils, namely animals L8 and L9 (Fig. 3E).

No obvious changes were detected in mRNA expression levels of the pro-inflammatory cytokines TNF-α, IL-12β, and IL-18, or in the inflammasome-related cytokine CASP1. A 10- to 25-fold increase in mRNA expression levels of IL-10 was observed at 5, 7, and 10 dpi in one of the two infected animals. Moreover, the levels of GM-CSF were upregulated from 5 dpi until the end of the experiment (18 dpi) in at least one out of the two infected sheep per time point, with its expression increasing up to 150-fold change in animal L15. No change in TGF-β expression was observed in any of the animals (Fig. 7F and H).

Protein levels of MX1, IFN-γ, and IL-1α, which were among the highly upregulated cytokines at the mRNA level in the tonsils, were assessed by ELISA, but no clear increase could be detected. Protein concentrations of ISG15, TNF-α, TGF-β, CXCL11, and CCL5 were also measured by ELISA. No increases were observed for TNF-α and CXCL11, while ISG15 and TGF-β concentrations were below detection limits. Only CCL5 showed a more than 10-fold increase in multiple infected animals compared to control animals (see Fig. S2 and S3).

### mRNA and protein expression in the cerebellum, thalamus, and medulla oblongata of LIV-infected sheep

**Fig 8:**
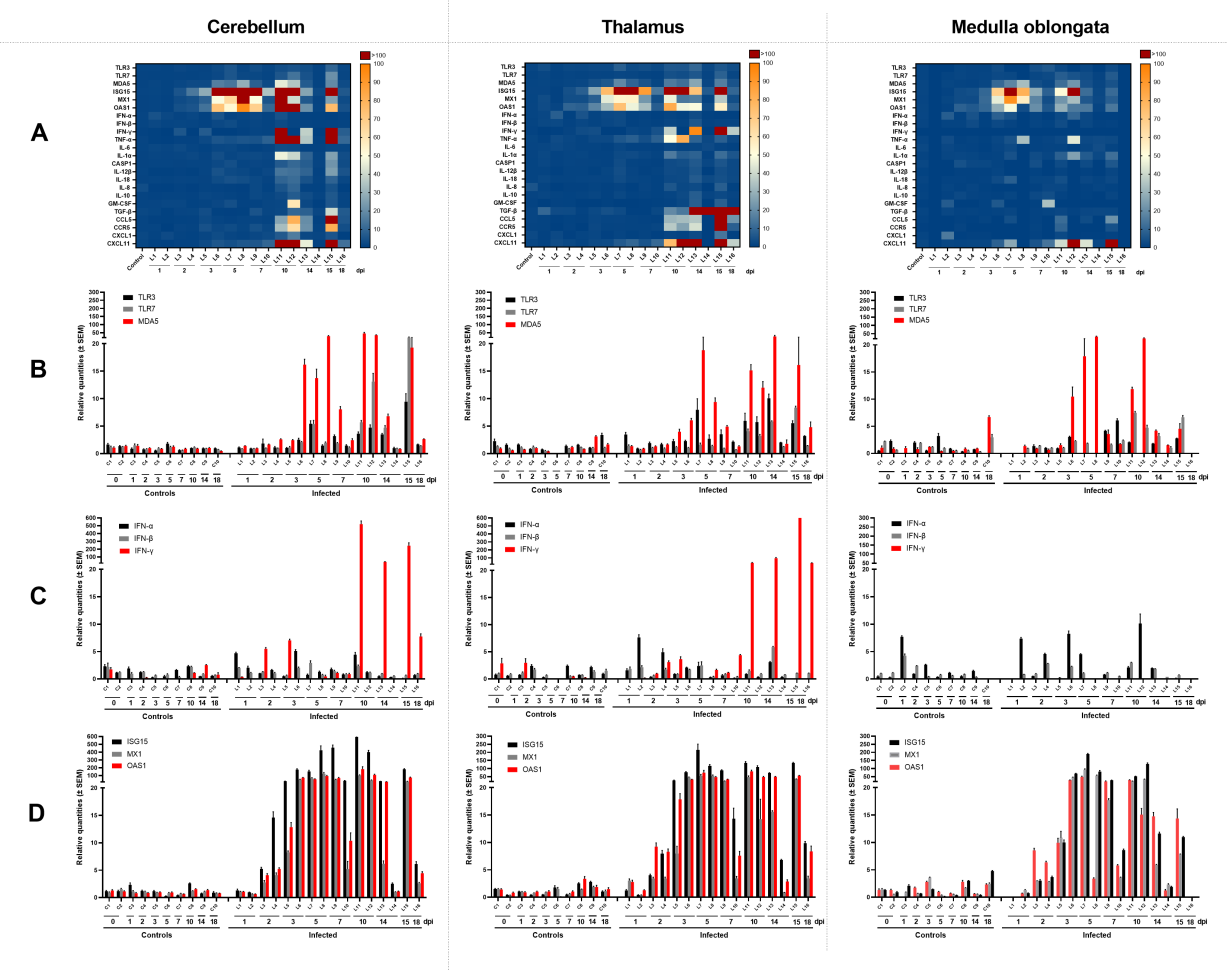
mRNA expression profiles of the selected genes in the cerebellum, thalamus, and medulla oblongata of 8-month-old sheep inoculated intradermally with LIV LI/31 strain at a dose of 10^5^ TCID_50_/sheep. (**A**) Heat map visualizations of mRNA expression changes in the selected genes. Fold changes in mRNA expression of (**B**) TLR3, TLR7, MDA5; (**C**) IFN-α, IFN-β, IFN-γ; and (**D**) ISG15, MX1, and OAS1 were determined by RT-qPCR, and relative quantification is depicted over time (at 1, 2, 3, 5, 7, 10, 14, 15, and 18 dpi). Target gene expression levels of infected animals were expressed relative to the average of the control group. Error bars represent technical replicates.

Among the analyzed PRR genes, MDA5 was upregulated in the cerebellum and thalamus of many sheep infected with LIV from 3 dpi onwards until 15 dpi. In the medulla oblongata, this upregulation was observed from 3 dpi to 10 dpi (Fig. 8B). The analysis of mRNA expression profiles of interferons showed no clear upregulation of type I IFNs (α and β), whereas an increase in IFN-γ mRNA was observed in the cerebellum and thalamus from 10 dpi until the end of the experiment in one out of two infected animals at each time point (Fig. 8C). Again, an overall and strong increase in the expression of ISGs was observed in the three brain tissues starting at 2 dpi (Fig. 8D). The cerebellum showed to be the brain area exhibiting the highest increase in mRNA expression levels of ISG15 and OAS1. At 10 dpi, a strong 400- and 600-fold increase in ISG15 expression was observed, and a 100- and 180-fold increase in OAS1 expression was detected (Fig. 8D).

**Fig 9:**
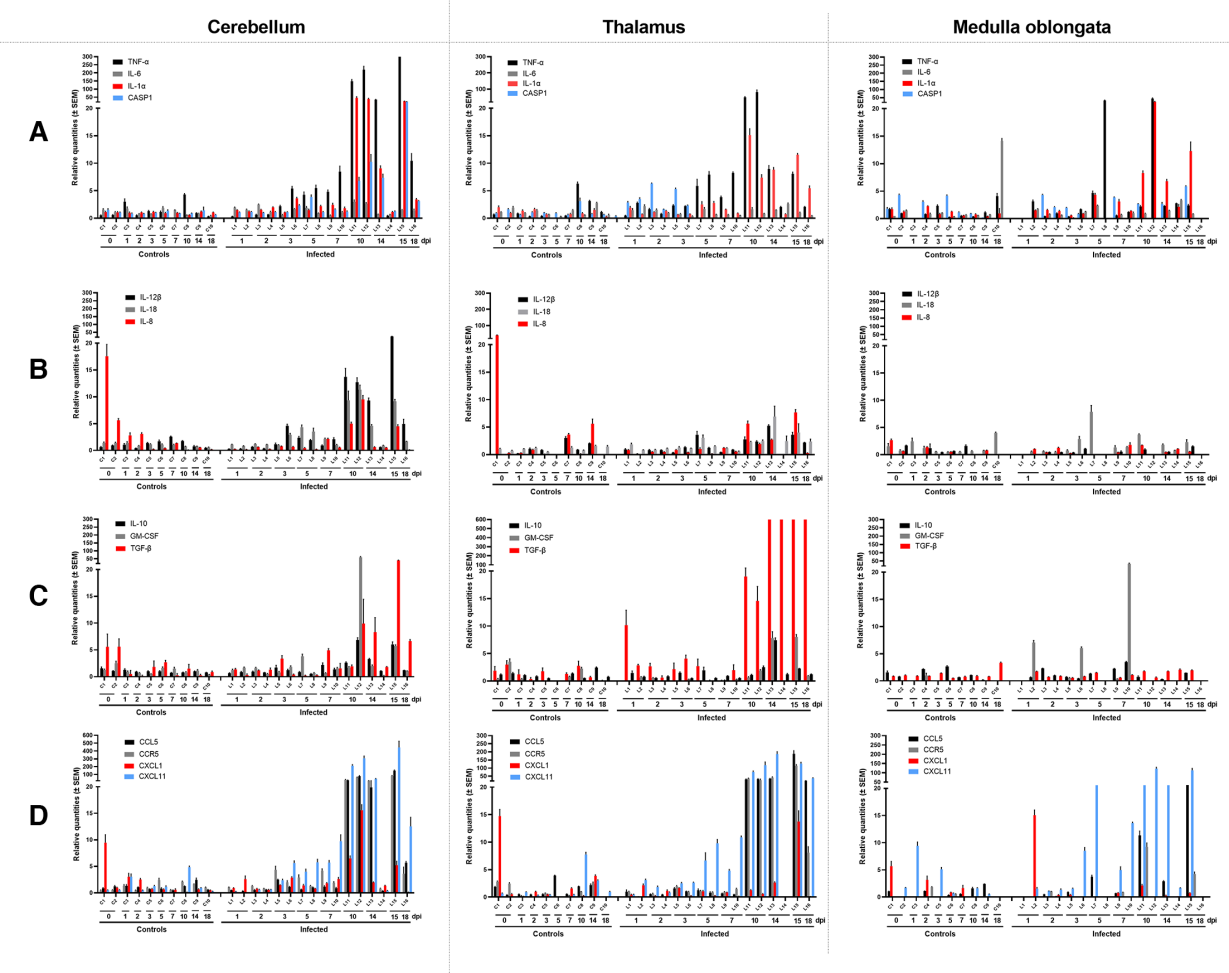
mRNA expression profiles of the selected genes in the cerebellum, thalamus, and medulla oblongata of 8-month-old sheep inoculated intradermally with LIV LI/31 strain at a dose of 10^5^ TCID_50_/sheep. Fold changes in mRNA expression of (**A**) TNF-α, IL-6, IL-1α, CASP1; (**B**) IL-12β, IL-18, IL-8; (**C**) IL-10, GM-CSF, TGF-β; and (**D**) CCL5, CCR5, CXCL1, and CXCL11 were determined by RT-qPCR and relative quantification and are depicted over time (at 1, 2, 3, 5, 7, 10, 14, 15, and 18 dpi). Target gene expression levels of infected animals were expressed relative to the average of the control group. Error bars represent technical replicates.

When analyzing the mRNA expression profiles of the pro-inflammatory, anti-inflammatory, and inflammasome-related cytokines in the brain, an increase in IL-1α (ranging from 5- to 45-fold) and TNF-α (ranging from 10- to 300-fold) was detected in the three brain parts analyzed starting at 10 dpi, but this was the most pronounced in the cerebellum (Fig. 8A). This organ also exhibited a moderate rise in CASP1, IL-12β, and IL-18. It is also noteworthy that a remarkable upregulation of TGF-β was observed in the thalamus from 10 dpi onwards (Fig. 9C), and that animals L12 and L15 were almost always those with the highest cytokine upregulation in the three brain areas (Fig. 8A). These animals displayed the highest viral RNA loads in the three brain tissues along with clinical symptoms.

A strong increase in mRNA expression levels of CCL5, CCR5, and CXCL11 was observed starting from 10 dpi in the cerebellum and thalamus of most animals. In the medulla oblongata, CXCL11 showed an increase from 5 dpi to 15 dpi, whereas CCL5 had only a rise at 10 and 15 dpi (Fig. 9D).

ELISA analysis showed that the high mRNA expression of IFN-γ observed in the cerebellum and thalamus starting 10 dpi was also reflected at the protein level (see Fig. S3). MX1, IL-1α, and CCL5 were upregulated both at the mRNA and protein levels in the brain tissues of animals exhibiting clinical symptoms (L8, L12, and L15). In contrast, TNF-α and TGF-β showed increased mRNA expression without corresponding increases at the protein level. ISG15 and CXCL11 protein concentrations were below the limit of detection (Fig. S2 and S3).

## DISCUSSION

Sheep play an important role in the epidemiology of TBEV and LIV, but their susceptibility to both viruses is different. TBEV infection is usually asymptomatic, whereas LIV causes a febrile illness in sheep that can progress to fatal encephalitis. Limited studies addressing the pathogenesis of TBEV and LIV in sheep and the associated immune responses have been undertaken. In order to provide first indications of viral spread and replication over time in various tissues and gain a better understanding of key components of the immune responses contributing to virus clearance, we carried out an *in vivo* infection study in sheep with TBEV and LIV.

Sheep were inoculated intradermally with a dose of 10^5^ TCID_50_ to mimic the natural route of infection (ticks); however, little data are available on the actual dose of TBEV or LIV that is delivered to sheep by a tick during natural infection. Lindblom et al. (39) reported that TBEV RNA copy numbers in ticks detached from humans ranged from less than 400 to 7.7 × 10⁶ per tick, likely depending on the tick species, viral strain, and feeding duration (39). Also, the absence of tick saliva, which is normally injected together with the virus by the tick and can modulate viral replication and host immune responses, might mean that our results deviate from what occurs in naturally infected sheep. In our experimental setting, TBEV-infected sheep displayed no fever or clinical signs throughout the study, whereas sheep infected with LIV exhibited a biphasic fever and clinical symptoms, including increased respiration rate, depression, reduced or suppressed appetite, lethargy, and head trembling. Our observations of TBEV or LIV infection in sheep align with those reported by Mansfield et al. (17) following subcutaneous infection (17).

The replication of arthropod-borne flaviviruses during natural infection or after experimental intradermal and subcutaneous inoculations starts at the inoculation site (mostly skin). Langerhans cells are a dendritic cell (DC) subpopulation in the epidermis, constituting the primary target of TBEV/LIV infection and replication. Infected DCs migrate to the draining lymph nodes, from where the virus spreads in the bloodstream, resulting in viremia (40–42). In agreement with this, our results show that viral RNA of TBEV and LIV was detectable in the skin and prescapular lymph nodes after 1 and 2 dpi, respectively. Additionally, TBEV and LIV loads in the prescapular lymph nodes seem to increase between 3 and 7 dpi, suggesting viral replication and further emphasizing the importance of the lymphatic system as an early site for viral replication (35).

Besides the prescapular lymph node, TBEV RNA detection was limited to low amounts in the spleen between 5 and 10 dpi, suggesting that TBEV can spread via the lymphatic system. No TBEV was detected in the blood, visceral organs, or brain. This suggests that TBEV replication is efficiently blocked by the innate immune response early after infection. The absence of viremia implies that the role of sheep in TBEV epidemiology is indirect and mostly related to hosting and enabling tick multiplication, rather than causing direct TBEV transmission to blood-feeding ticks. Despite the absence of productive replication in organs other than the prescapular lymph nodes, a strong antibody response was observed at 7 dpi, which aligns with findings from previous studies (17, 43).

The limited TBEV replication in the prescapular lymph node and reduction in viral load after 7 dpi correlated with 5- to 40-fold changes in the expression profiles of ISG15, MX1, OAS1, IL-1α, and CXCL11 in the prescapular lymph nodes at 5 and 7 dpi. The upregulation of ISG15, MX1, and OAS1 is consistent with their involvement in the interferon-induced antiviral response. ISG15 appears to play a role in the immune responses *in vitro* against several flaviviruses. ISG15 was shown to be upregulated in human neuronal/glial cultures infected with TBEV and in human fibroblasts following Zika virus (ZIKV) infection. Studies in mouse macrophage-like cell line RAW264.7 reported an upregulation of ISG15 upon DENV or WNV infection, and its overexpression has been associated with the inhibition of JEV replication in human medulloblastoma cells (44–47). MX1 is responsible for the inhibition of viral replication by targeting the viral nucleocapsid and preventing its import into the nucleus (48). Its expression in human neuronal/glial cultures infected with TBEV was shown to be activated almost immediately post-infection, at 7 hpi, and to increase progressively until 14 dpi (45). OAS1 degrades viral RNA by activating RNase L (49–51), making it a key element of the antiviral intracellular innate immune response in mammalian cells. Humans carrying OAS single nucleotide polymorphism (SNP) were reported to be predisposed to severe forms of TBEV (52, 53). Despite the upregulation of the ISGs, we surprisingly did not observe a clear upregulation of type I interferons in the prescapular lymph nodes upon TBEV infection. The most likely explanation is that TBEV blocked type I IFN signaling and that ISGs expression was activated by an alternative mechanism independent of the IFN-mediated pathway, as reported for WNV infection (54–56). Next to ISGs and in agreement with previous studies, we observed an upregulation of IL-1α and CXCL11 (45, 57, 58). IL-1α is a pro-inflammatory cytokine whose expression induces the production of chemokines and leads to the infiltration of neutrophils followed by monocytes (59, 60). The analysis of mRNA expression profiles of cytokines and chemokines in the brain of TBEV-infected mice showed an enhancement of IL-1α mRNA expression from 6 dpi onwards (57). CXCL11 is an IFN-γ-inducible chemokine that plays a role in the trafficking of leukocytes, mainly activated CD4+ Th1 (T helper 1) cells, CD8+ T cells, and NK cells (61). Elevated levels of CXCL11 were observed in the CSF of patients infected with TBEV and showed to be highly upregulated in human neurons and astrocytes (45, 58). Overall, it appears that the fast and moderate activation of multiple interferon-stimulated genes, together with some crucial pro-inflammatory cytokines and chemokines, is sufficient to efficiently block TBEV replication and spread early after inoculation, thereby preventing systemic spread in sheep.

Concurrently with its detection in the prescapular lymph nodes, LIV RNA was initially detected in the bloodstream at 2 dpi with peak viremia at 5 dpi. Previous studies also reported maximum concentrations of LIV in serum samples at 4 or 5 dpi (34). Peak viremia dropped by 7 dpi, correlating with the first detection and subsequent increase of neutralizing antibodies from 7 dpi onwards. Our results regarding the timing of the first detection of anti-LIV antibodies are in line with those reported by Mansfield et al. (17). Next to LIV detection in the bloodstream, the spleen and tonsils tested positive for LIV RNA from 2 and 3 dpi onwards, respectively, and remained positive until the end of the experiment. Moreover, LIV viral loads in these organs increased between 3 dpi and 7 dpi, suggesting replication and tropism of LIV for the lymphoid organs. This seems plausible since the extensive replication of LIV within the lymphatic tissues was previously described (35). Next to the lymphoid organs, kidneys and lungs became LIV RNA positive at the time of peak viremia, whereas the liver became positive already at 3 dpi. These results suggest a blood-borne dissemination of LIV to the visceral organs. However, there is little evidence of local replication in the visceral organs.

Besides the spread of LIV to the lymphoid and visceral organs, neuroinvasion occurred, as supported by the detection of LIV RNA from 5 dpi onwards in all tested CNS areas. The mechanism of entry of neurotropic flaviviruses into the CNS is still not well defined (62, 63) and includes bypassing the host barrier through the hematogenous route, the trans-neural route, and across unprotected areas of the CNS (i.e., circumventricular organs [CVOs]) (64–66). Viral entry via the hematogenous route implicates direct infection of endothelial cells of CNS blood vessels (transcellular passage), induced blood–brain barrier (BBB) breakdown (paracellular passage), and/or the hijacking of migrating peripheral leukocytes (Trojan horse mechanism) (64, 65). Our study results show that peak detection of LIV in the serum and its initial detection in all tested brain areas occurred simultaneously at 5 dpi. This observation might potentially support LIV entry into the CNS via the hematogenous route, although additional experiments remain necessary to provide irrevocable proof and clarify the exact mechanism of entry. Several viruses, such as rabies and poliovirus, infiltrate the CNS through trans-neural pathways via peripheral and olfactory nerves; however, information on flavivirus infiltration through this route is limited (3, 64, 65). Recently, Fingerhood et al. (67) reported for the first time the detection of LIV antigen in the brachial plexus of a naturally LIV-infected dog and suggested the peripheral nerves as a potential route of LIV entry into the central nervous system (67). Finally, some viruses target the areas in the brain lacking the protection of BBB, such as the choroid plexus (65). Our results indicate stable LIV viral loads in the olfactory bulb over time and a moderate increase of LIV viral loads in the trigeminal ganglion and the choroid plexus between 5 and 10 dpi. This may suggest that these entry routes may not play an important role in LIV neuropathogenesis in sheep.

Our findings show that LIV viral loads peaked at 10 dpi in almost all brain areas, except in the animal that was humanely euthanized earlier due to the presence of neurological symptoms. The highest viral loads of LIV at 10 dpi were found in the medulla oblongata and the pons, followed by the cerebellum. This is in agreement with previous studies reporting a predilection of multiple flaviviruses, including JEV, WNV, TBEV, and LIV, for the brain stem (medulla and pons) and the cerebellum (68–70). These parts of the brain are associated with control of movements, coordination, and equilibrium, which may explain the occurrence of tremors, motor incoordination, and ataxia in animals infected with LIV (69, 71). In the hypothalamus and the thalamus, the highest viral loads were detected at 15 dpi in the animal that was euthanized earlier. This animal was dyspneic, louping, and recumbent. The hypothalamus and thalamus are implicated in perception, movement, and vital body functions (71), and their infection might have likely contributed to these symptoms. The thalamus has been reported as an important tissue for JEV replication in mice and pigs, and thalamic lesions are the most commonly reported abnormality in brain imaging of patients with TBEV or JEV infection and dogs with flavivirus infection (72–77).

Compared to TBEV, sheep inoculation with LIV showed to initiate a stronger antiviral innate immune response in the prescapular lymph nodes, as indicated by the high upregulation of ISG15, MX1, and OAS1 mRNA in all animals from 2 dpi to 7 dpi. This coincided with the initial detection of LIV RNA in this tissue at 2 dpi. The strong induced expression of the studied ISGs, however, did not seem to control LIV replication in the prescapular lymph nodes, where LIV viral loads increased between 2 and 7 dpi. This suggests that the innate immune response was insufficient to stop LIV replication. The increase in viral loads despite the induction of a strong interferon-stimulated gene response could even suggest that the overexpression of certain ISGs such as ISG15 might enhance viral replication mainly via the ISGylation process as previously reported for ZIKV, hepatitis B virus (HBV), and hepatitis C virus (HCV) (78–81). Concurrently, the expression profiles of TLR7 and MDA5 in the prescapular lymph nodes changed between 5- and 15-fold from 3 dpi to 5 dpi. TLR7 and MDA5 belong, respectively, to the TLRs and RLRs (RIG-I-like receptors) families and are involved in detecting viral RNA. Both TLRs and RLRs mediate flavivirus recognition in mammalian cells and initiate antiviral immune responses by inducing the secretion of type I interferons and producing pro-inflammatory cytokines, such as interleukin 1β (IL-1β) and IL-18 (54, 82, 83). Our results suggest that TLR7 and MDA5 signaling pathways are likely involved in sensing LIV, similarly to other flaviviruses.

With the exception of animal L15, the expression profiles of IFN-α, IFN-β, IFN-γ, TNF-α, and GM-CSF in the prescapular lymph nodes of LIV-infected sheep did not show remarkable changes. Animal L15 demonstrated a strong increase in IFN-α mRNA expression (>120-fold) and an approximately 10-fold change in TNF-α and GM-CSF mRNA at 15 dpi. Moreover, an increase in the expression levels of IL-8, IL-6, and IL-1α was also observed, particularly at 7 and 10 dpi, in the prescapular lymph nodes of the animals that manifested fever and clinical signs. In line with our results, previous studies on other flaviviruses reported a correlation between the clinical signs and the upregulation of IFN-α, TNF-α, GM-CSF, IL-8, IL-6, and IL-1α (54, 84–86).

A similar pattern of upregulation of different PRRs (TLR3 and TLR7) and ISG (MX1, OAS1, and ISG15) mRNAs, as in the prescapular lymph nodes, was observed in the tonsils of LIV-infected sheep. The main difference with the prescapular lymph nodes was a clear increase in both type I and type II interferon mRNA expression, with the tonsils being the only tissue in which this was observed. Additionally, the levels of IL-10, GM-CSF, and CCR5 in the tonsils showed a consistent increase from 5 dpi onwards until the end of the experiment. All these cytokines are key players of the innate immune response and were reported to be upregulated in other flaviviral infections (54, 84–86). However, despite the strong upregulation of multiple cytokines, the viral load in the tonsils started to decrease only after LIV-specific antibodies were detected in the blood and a marked increase in IFN-γ expression was observed between 7 and 10 dpi.

In naturally LIV-infected sheep, the regions of the CNS with the most severe lesions are the medulla, pons, cerebellum, and thalamus (7). These regions also showed the highest LIV RNA loads in our study and were most likely subject to local virus replication and associated immune responses. Based on our results, MDA5 mRNA expression was upregulated in the cerebellum and thalamus from 3 dpi to 15 dpi and in the medulla oblongata from 3 dpi to 10 dpi. Overall, ISG expression increased in these three brain regions starting at 2 dpi, with the cerebellum showing the highest increase in ISG15 and OAS1 mRNA levels at 10 dpi. In line with our results, MX1 has been shown to be upregulated in the pons and the medulla of sheep experimentally infected with LIV (17, 45). The upregulation of MDA5, ISG15, or OAS1 in brain tissues or brain-derived cell cultures following flaviviral infection was also previously reported (9, 45–47, 72, 87). In the medulla, the induction of other pro- and anti-inflammatory cytokines remained more limited compared to the cerebellum and the thalamus. In these areas, a strong increase in IFN-γ expression levels was also observed from 10 dpi onwards in one of the two infected animals per time point. In agreement with our results, an upregulation of IFN-γ was found at post-mortem in the brain of sheep experimentally infected with LIV (17). Furthermore, a remarkable increase in TGF-β was observed specifically in the thalamus. Finally, the expression of several of the studied chemokines was also upregulated, with CCR5, CCL5, and CXCL11 expression increasing from 10 dpi onwards in the cerebellum and thalamus, while only CXCL11 showed an increase in the medulla oblongata. TGF-β is mainly produced by microglia and astrocytes following brain injury. High levels of this cytokine were associated with Alzheimer’s disease and ischemic stroke (88), but little information is available on its implication in flavivirus infection. A high induction of CCR5/CCL5 was reported in ZIKV-infected hBMECs (human brain microvascular endothelial cells), in TBEV-infected human brain pericytes, and in the brain of TBEV and WNV-infected mice (89–93). In agreement with our results, previous studies reported an upregulation of CXCL11 in mouse brain infected with WNV, in the CSF of TBEV patients, and in JEV-infected SK-N-MC cells (58, 94–96). Altogether, the results indicate that different levels of immune reactions are necessary in different parts of the CNS to control LIV replication.

Sheep L15, which needed to be humanely euthanized due to severe neurological manifestations, showed the highest upregulation of chemokines and several cytokines, such as IFN-γ, TNF-α, IL-1a, CASP1, IL-12β, and TGF-β of all inoculated sheep, suggesting that the cytokine storm in different parts of the brain contributed to these symptoms. This had been already observed for several flaviviruses in mice, where an uncontrolled immune response correlated with the induction of encephalitis. During TBEV infection, for instance, pro-inflammatory mediators play a major role in attracting immunocompetent cells to the CNS, which can in turn activate apoptosis signaling pathways in neurons, leading to neuronal death or disruption of the BBB (57, 97–100). Based on our results, the upregulated cytokines in the brain in response to LIV infection are primarily pro-inflammatory, with the exception of CASP1, GM-CSF, and TGF-β, which are inflammasome-related and anti-inflammatory cytokines, similar to what was reported by previous studies following replication of flaviviruses in the CNS of humans and mice (45, 54, 57, 58, 101–104). This combination of immune mediators with opposite functions strongly suggests that a localized dysregulation of the immune response in the neuronal tissue may be one of the mechanisms underlying LIV pathogenesis in sheep.

Some cytokines showing strongly increased mRNA levels in brain tissues of animals with clinical symptoms, namely IFN-γ, MX1, IL-1α, and CCL5, also showed corresponding increases at the protein level, indicating a biologically relevant protein response that may contribute to neuropathogenesis. For TNF-α and TGF-β, whose mRNA levels were also upregulated in the brain tissues of clinically affected animals, such correspondence at the protein level could not be found. Several biological factors can be responsible for this discrepancy, including post-transcriptional regulation, protein degradation in tissue samples, and/or temporal delays between transcription and translation. Nevertheless, it cannot be excluded that technical factors also impacted the obtained results. Commercial ELISA kits or other approaches allowing cytokine quantification at the protein level in sheep are limited. The kits used in this study are research-use-only kits not specifically validated for protein quantification in all tissue types analyzed, which may affect assay sensitivity. For example, protein levels of ISG15, TGF-β, and CXCL11 were below the lower limit of detection in some tissues, meaning that they could not be assessed. Cytokine protein concentrations were also measured in serum, where no difference in any of the tested cytokines (MX1, ISG15, TNF-α, IL-1α, CCL5, TGF-β, and CXCL11) was observed between control and infected animals (see Fig. S4). This is in line with the view that cytokines primarily function as local rather than systemic mediators.

The results described above provide valuable temporal data on viral dissemination and the role of specific immune responses to TBEV and LIV infection of sheep, certainly in a field that has only been little studied for the moment. Due to logistical, financial, and ethical constraints related to performing studies in large animals, only two animals per time point could be studied in the current experiment, meaning that future studies will be necessary to further explore some of the findings and perform more in-depth analyses of the induced immune responses.

### Conclusion

Different disease outcomes were observed upon intradermal infection of sheep with TBEV and LIV. Clinical symptoms were absent in TBEV-infected sheep, and viral detection was confined to the skin, the prescapular lymph nodes, and the spleen. Only a weak innate antiviral response was induced in the prescapular lymph nodes, which, together with the humoral immune response, seemed sufficient to counteract TBEV replication and spread early after infection. In contrast, a disseminated infection was observed in sheep infected with LIV, with a tropism for lymphoid tissues and the CNS, leading to the induction of clinical neurological signs in some animals. The induction of a strong innate antiviral response in the prescapular lymph nodes seems inefficient to stop LIV replication early upon infection and to prevent viremia and invasion of the CNS. It was only after the onset of the humoral immune response, and possibly the cell-mediated immune response, that viral loads in the prescapular lymph nodes and tonsils decreased. While further studies are necessary to substantiate these findings, entry into the CNS occurred likely via the hematogenous route based on the correlation of the simultaneous first detection of LIV RNA in all tested CNS regions with the peak of viremia, but the contribution of a peripheral trans-neural route could not be excluded. Finally, LIV demonstrated a predilection for the brain stem, the cerebellum, and the thalamus, and its replication in these tissues induced a strong upregulation of ISGs, chemokine, and pro-inflammatory cytokine responses. Our data thus suggest that LIV has evolved more efficiently than TBEV to circumvent and overcome the induced immune responses in this species.

## MATERIALS AND METHODS

### Virus isolates

TBEV Neudoerfl strain and LIV LI/31 strain, provided respectively by Dr. Vanessa Suin (previously at Sciensano, presently at Clinique St-Pierre Ottignies) and Dr. Mara Rocchi (Moredun Research Institute), were passaged twice in Baby Hamster Kidney-21 (BHK-21) cells before use in the animal experiment. This resulted in virus stocks with a titer of 10^7.49^ TCID_50_/mL for TBEV and 10^7.41^ TCID_50_/mL for LIV.

### Experimental design

The experimental protocol was approved by the ethical committee of Sciensano (20200515-01) and was conducted in BSL3 animal facilities (Sciensano, Machelen, Belgium). A total of 42 8-month-old Texel sheep originating from a small ruminant lentivirus (SRLV)-free herd were used in this study. To ensure their negative infection status, sheep were tested for TBEV/LIV and *A. phagocytophilum* (known to enhance LIV pathogenicity [7]) by RT-qPCR and ELISA prior to the experiment. The animals were allowed to adapt to the BSL3 facility for a week before the experiment and were then randomly divided into three groups. The first and the second groups comprised each 16 sheep and were inoculated intradermally in the neck with a final dose of 10^5^ TCID_50_ of TBEV or LIV, respectively. The titers were confirmed by back titration of the inoculum. The third group (*n* = 10) was inoculated with minimum essential medium (MEM, Gibco) as a control group. The three groups were housed in separate pens. Rectal temperature and clinical signs were monitored daily. Each animal was scored for general condition, appetite, respiratory, and neurological signs using the scoring card provided in Table S1, and predefined humane endpoints were applied. Two mock-infected sheep were euthanized on the day of infection (0 dpi). At 1, 2, 3, 5, 7, 10, 14, and 18 dpi, two TBEV- and two LIV-infected sheep and one control animal were euthanized. Blood, multiple tissues (lung, kidney, liver, prescapular lymph nodes, spleen, tonsils, hypothalamus, thalamus, cerebellum, cerebrum, choroid plexus, pons, medulla oblongata, spinal cord, olfactory bulb, and trigeminal ganglion), and skin biopsies at the site of inoculation were collected at each time point. To provide additional repeats per time point to study viremia and virus neutralization, blood was also collected from three and two predefined randomly selected infected and control animals, respectively, that were euthanized at later time points.

### Detection of TBEV and LIV by RT-qPCR

With the exception of the prescapular lymph nodes, tonsils, and skin samples, approximately 0.5 cm^3^ of tissue was homogenized in 1 mL of phosphate-buffered saline (PBS) with zirconia beads (0.1 mm) using a tissue lyser (TissueLyser II, QIAGEN) for 4 min at 25 Hz. The weight of each piece of tissue was determined and taken into consideration for the conversion to TCID_50_/g. Prescapular lymph nodes and tonsils were homogenized in 1 mL of PBS using two stainless steel beads (5 mm, Qiagen) and a tissue lyser for 4 min at 25 Hz. After centrifugation for 1 min at 10,000 rpm, 100 µL of homogenate was collected for RNA extraction. Frozen skin was homogenized in 1 mL of Trizol using the ULTRATURRAX homogenizer (IKA LABORTECHNIK). After the addition of 200 µL of chloroform, samples were centrifuged at 14,000 rpm for 15 min, and the aqueous layer containing the RNA was harvested. RNA from skin and tissue homogenates was extracted using the RNeasy kit (Qiagen) following the manufacturer’s protocol. Five microliters of eluted RNA was used to assess the presence of TBEV and LIV RNA by RT-qPCR as described by Schwaiger et al. (105). The efficiency and the detection of this RT-qPCR for both viruses were checked beforehand. All samples were also tested for the presence of β-actin as an extraction control. In each run, negative extraction and negative and positive amplification controls were also included. All RT-qPCRs were carried out on a LightCycler 480 Real-Time PCR System (Roche, Basel, Switzerland) using the AgPath-ID One-Step RT-PCR Reagents (Applied Biosystems, Thermo Fisher) as instructed by the manufacturer. A standard curve was generated by testing a 10-fold serial dilution of TBEV and LIV second passage stocks in RT-qPCR and was used to convert Ct values in equivalent viral loads as TCID_50_/mL or TCID_50_/g.

### Plaque reduction neutralization test

The presence of neutralizing antibodies against TBEV and LIV was tested by plaque reduction neutralization test (PRNT). For this purpose, sheep sera were decomplemented (30 min at 56°C), 5-fold diluted, and then serially 2-fold diluted (from 1/5 to 1/640) in Dulbecco’s modified Eagle medium (DMEM, Gibco) in 96-well plates. Subsequently, between 20 and 50 plaque-forming units of virus were added to each serum dilution and incubated for 1 h at 37°C. The virus-serum mix was afterward added to a 95% confluent monolayer of Vero cells and incubated for 72 h at 37°C. Cells were then fixed with methanol for immunofluorescence staining. A primary LIV-cross reactive anti-TBEV NS1 antibody (R&D systems, Bio-Techne Ltd.) and a secondary Alexa Fluor-conjugated goat anti-mouse IgG antibody (Invitrogen, Thermo Fisher) were used for the detection of viral antigens. All sera were tested in duplicate, and the number of plaques per well was counted under a fluorescence microscope (Leica Microsystems). Wells in which the number of plaques was reduced by 50% or more compared to the number of plaques in control wells were considered neutralized. Based on literature (106–110), the cut-off for positivity was fixed at a titer of 1/10.

### Titration of TBEV and LIV in tissue samples

In order to assess the presence of infectious virus and quantify it in the prescapular lymph nodes, tonsils, thalamus, cerebellum, medulla oblongata, and pons, homogenates of these tissues were prepared as described above and then serially diluted 10-fold to a final dilution of 10^−3^. One hundred microliters of all dilutions were afterward added in octuplicates to 90% confluent BHK-21 cells grown in 96-well plates. The inoculum was removed after 2 h of incubation, and cells were washed and incubated with fresh MEM medium for 72 h at 37°C. The plates were then placed at −80°C, freeze-thawed, and a second passage was carried out by transferring 100 μL of supernatants from the first passage to 90% confluent BHK-21 cells. After 2 h of incubation at 37°C, the inoculum was replaced with fresh medium, and the cells were incubated for 72 h of incubation at 37°C. Cells were then fixed with methanol and subsequently stained as described above. Finally, cells were visualized under a fluorescence microscope, and the titer was determined using the Reed–Muench method.

### cDNA synthesis and preamplification for cytokine quantification

Genomic DNA contamination was removed by treating the extracted RNA with Turbo DNase (Thermo Fisher Scientific, Waltham, MA, USA) following the manufacturer’s instructions. The concentration and the purity of RNA samples were determined using a Nanodrop 2000 instrument (Thermo Scientific), and RNA was converted to cDNA using the M-MLV Reverse Transcriptase kit (Invitrogen, Thermo Fisher Scientific, Waltham, MA, USA). In order to obtain sufficient cDNA amounts to study cytokine and chemokine gene expression in target organs, a preamplification step was performed using the Taqman PreAmp Master Mix (Applied Biosystems, Thermo Fisher Scientific, Waltham, MA, USA) according to the manufacturer’s instructions. A mix of primers targeting a selection of reference genes (housekeeping genes) and genes of interest (Table S2) was prepared at a final concentration of 0.1 µM per primer. The preamplification program consisted of an initial denaturation step of 10 min at 95°C, followed by 14 cycles of amplification at 95°C for 5 s and 4 min at 60°C. One selected sample of prescapular lymph nodes, tonsils, medulla oblongata, cerebrum, cerebellum, and thalamus was tested for all reference and target genes before and after preamplification to check for preamplification uniformity, as follows. Normalized Ct values of the different target genes to the reference genes before (cDNA) and after preamplification (PreAmp) were calculated according to the following formulas: ∆Ct [cDNA] = Ct [cDNA target gene] − Ct [cDNA reference gene]. ∆Ct [PreAmp] = Ct [PreAmp target gene] − Ct [PreAmp reference gene]. The ∆∆Ct was subsequently determined from the difference of the two ∆Cts (∆∆Ct = ∆Ct [PreAmp] − ∆Ct [cDNA]). PreAmp uniformity was considered satisfactory when ∆∆Cts were within the range −1.5 to +1.5.

### mRNA expression by qPCR and relative quantification

Primers and probes for one reference gene (ACTB) and eight genes of interest (IFN-α, IFN-β, IFN-γ, TNF-α, IL-8, IL-10, GM-CSF, and TGF-β) were previously described by Toussaint et al. (111) and by Michiels et al. (112), respectively (111, 112). Primer and probes for three reference genes (GAPDH, B2M, and YWHAZ) and 15 targets of interest (IL-1α, IL-6, IL-18, IL-12β, CCL5 (RANTES), CCR5, CXCL1, CXCL11, CASP1, OAS1, MX1, MDA5, ISG15, TLR3, and TLR7) were designed in this study using Primer3 (ELIXIR, v.4.1) (or PrimerQuest tool from IDT, Coralville, USA) and based on sheep sequences available in the NCBI GenBank. RT-qPCR efficiency was determined for all the genes designed in this study using the standard curve method. All primers and probes were purchased from Integrated DNA Technologies (IDT, Coralville, USA) and are listed in Table S2. All RT-qPCRs were run on a LightCycler 480 Real-Time PCR system (Roche, Basel, Switzerland) using SsoAdvanced Universal Probes Supermix (Bio-Rad). A duplex RT-qPCR was performed when possible. Each qPCR reaction consisted of 10 µL of Supermix, 1 µL of primers/probe mix (final concentration of 0.5 µM and 0.2 µM, respectively), 4 µL (or 3 µL if duplex reaction) of RNase-free water, and 5 µL of preamplified cDNA. All samples were tested in duplicate using the following program: 95°C for 3 min, followed by 45 cycles of 15 s at 95°C, and 45 s at 60°C. The appropriate reference genes for normalization were determined beforehand for each target organ using the GNorm method. B2M and GAPDH were identified as the most suitable reference genes for the prescapular lymph nodes, while ACTB and GAPDH were selected for the tonsils. The most suitable reference genes for the cerebellum were YWHAZ and GAPDH. For the thalamus, YWHAZ and ACTB were identified as the best reference genes. Finally, GAPDH, ACTB, and YWHAZ were selected for the medulla oblongata. The normalization and quantification of relative expression levels were performed using qBase PLUS (Biogazelle, v.3.2). Target gene expression levels of infected animals were expressed relative to the average of the control group. GraphPad Prism 9 (San Diego, CA, USA) was used to make all bar charts.

### Detection of selected innate immune proteins by ELISA

To complement the mRNA expression results, commercial sheep ELISA kits from Clinisciences (ISG15, MX1, IL-1α, CCL5, and CXCL11) and Antibodies.com Europe AB (TNF-α and TGF-β) were used to quantify the protein concentrations in the tonsil, cerebellum, thalamus, and medulla oblongata of LIV-infected animals at 1, 5, 10, and 15 dpi and of control animals at 1, 5, and 10 dpi. Serum samples from all TBEV-infected, LIV-infected, and control animals were also analyzed for these proteins. The assays were performed following the manufacturer’s instructions. Protein concentrations were determined using standard curves. Concentrations in tissue homogenates were normalized to tissue weight and expressed as pg/g or ng/g, while concentrations in serum were expressed as pg/mL or ng/mL, depending on the assay. The upper limit of quantification (ULOQ) and lower limit of quantification (LLOQ) were defined based on the upper and lower limits of the standard curve range in each assay. Finally, IFN-γ was assessed in the same tissues and serum using the ID Screen Ruminant IFN-γ commercial kit. Sample reactivity to IFN-γ was determined by comparing optical density (OD) values of samples to those of positive and negative controls. ELISA assays in which the majority of measured values were below the detection limit were not considered for comparison with mRNA expression results. All results are presented in the supplementary materials.

### Statistical analysis

Mann-Whitney U tests were performed using GraphPad Prism 9.4.1 to assess the differences in temperature and clinical scores between the baseline (day 0 for temperature and day 1 for clinical scores) and each other time point within the LIV-infected group. *P*-values < 0.05 were considered significant.

## Data Availability

The authors confirm that the data sets generated during this study are included within the article and its supplemental material.

## SUPPLEMENTAL MATERIAL

Supplemental material jvi.00651-26-s0001.docx Tables S1 and S2; Fig. S1 to S4.

ASM does not own the copyrights to Supplemental Material that may be linked to, or accessed through, an article. The authors have granted ASM a non-exclusive, world-wide license to publish the Supplemental Material files. Please contact the corresponding author directly for reuse.
