# Supplementary material for: Tissue tropism and mRNA expression profiles of selected innate immunity-related genes following experimental tick-borne encephalitis virus and louping ill virus infection of sheep
Source: J Virol. 2026 Jun 25;100(7):e00651-26. doi: 10.1128/jvi.00651-26 (PMC13386917; doi:10.1128/jvi.00651-26)
Supplement: Supplemental material — Tables S1 and S2; Fig. S1 to S4. [file jvi.00651-26-s0001.docx]

**Table S 1** Scoring card used for the monitoring of the clinical signs of sheep

|  |  |  |  |  |  |  | **Neurological signs** | | | |
| --- | --- | --- | --- | --- | --- | --- | --- | --- | --- | --- |
|  |  |  |  |  |  |  | Trembling | | Motor incoordination | Leaping |
|  |  | **Nasal Discharge** | **Coughing** | **Breathing** | **General** | **Appetite** | Head | Post-M |  |  |
| Score | **0** | Normal | None | Normal | Normal | Normal | **0= none** | | | |
|  | **1** | Mild mucous | Spontaneous; 1-3/5 min | Increased respiration rate | Slight depression | Reduced |  |  |  |  |
|  | **2** | Marked Mucous | > 3/5min | Abdominal breathing | Lethargy | No appetite | **1= present** | | | |
|  | **3** | Purulent | / | Gasping | Down | / |  |  |  |  |

**Table S 2** Primer/probe sequences used for the detection of selected reference genes and cytokines

|  | **Description** | **Primers/probes** | **Sequence (5'-3')** | **Amplicon size** | **Efficiency (%)** | **source** |
| --- | --- | --- | --- | --- | --- | --- |
| **Reference genes** | | | | | | |
| ACTB | β-actin | ACTB_F | CAGCACAATGAAGATCAAGATCATC | 131 | 96 | (Toussaint et al., 2007) |
|  |  | ACTB_R | CGGACTCATCGTACTCCTGCTT |  |  |  |
|  |  | ACTB_P | TCGCTGTCCACCTTCCAGCAGATGT |  |  |  |
| GAPDH | Glyceraldehyde 3-phosphate dehydrogenase | GAPDH_F | CCCTTCATTGACCTTCACTACA | 108 | 95 | Primer Questool |
|  |  | GAPDH_R | TCCATTGATGACGAGCTTCC |  |  |  |
|  |  | GAPDH_P | ACGGCACAGTCAAGGCAGAGAA |  |  |  |
| B2M | β2-microglobulin | B2M_F | ACCTTGGGCCCTACTTGTCCA | 85 | 108 | primer 3 |
|  |  | B2M_R | TGTCTTCCCCACCTCCACGA |  |  |  |
|  |  | B2M_P | GCAGGTATCTGTGGTGGAGGCGCTGGCA |  |  |  |
| YWHAZ | Tyrosine 3-monooxygenase activation protein zeta polypeptide | YWHAZ_F | TCAGTCACAGCAAGCATACC | 98 | 105 | Primer Questool |
|  |  | YWHAZ_R | CAGAGAAGTTAAGGGCCAGAC |  |  |  |
|  |  | YWHAZ_P | TGCAACCAACACATCCTATCAG ACTGG |  |  |  |
| **Genes of interest** | | | | | | |
| IFN-α | Interferon alpha | IFN-α_F | TCGACAACTGAGGAGGGTCT | 161 | 105 | (Michiels et al., 2021) |
|  |  | IFN-α_R | CCTCTGTGCTGAAGAGCTGG |  |  |  |
|  |  | IFN-α_P | TGGGTGGCAGCCAGTTGCAG |  |  |  |
| IFN-β | Interferon beta | IFN-β_F | GATGAAGCAAGCACAGCAGT | 181 | 107 |  |
|  |  | IFN-β_R | TGGCTCCAGACGATTCATCT |  |  |  |
|  |  | IFN-β_P | CACCAGAGACTTCTCCAGCA |  |  |  |
| IFN-γ | Interferon gamma | IFN-γ_F | CCATAACACAGGAGCTACCGAT | 91 | 110 |  |
|  |  | IFN-γ_R | GAGCAGTAAAGCTAAGAAGGAGC |  |  |  |
|  |  | IFN-γ_P | ACTACTCCGGCCTAACTCTCTCCT |  |  |  |
| TNF-α | Tumor necrosis factor alpha | TNF-α_F | ACGAACCCATCTACCAGGGA | 77 | 95 |  |
|  |  | TNF-α_R | TTCCGGCAGGTTGATCTCAG |  |  |  |
|  |  | TNF-α_P | AGCTGGAGAAGGGAGATCGCCT |  |  |  |
| IL-8 | Interleukine-8 | IL-8_F | CATTCCACACCTTTCCACCC | 124 | 108 |  |
|  |  | IL-8_R | GGGGTCTAAGCACACCTCTTT |  |  |  |
|  |  | IL-8_P | TGAGAGTGGGCCACACTGCG |  |  |  |
| IL-10 | Interleukine-10 | IL-10_F | ACCTGTGTTTAAGCTGTTTCCA | 249 | 109 |  |
|  |  | IL-10_R | AGAGGGCAGTCAGGGAAAAC |  |  |  |
|  |  | IL-10_P | TGCAGTTTCCATTCCAAGCC |  |  |  |
| GM-CSF | Granulocyte macrophage colony stimulating factor | GM-CSF_F | ACTGTGGTCTGCAGCTTCTC | 109 | 101 |  |
|  |  | GM-CSF_R | TGCTGTCGTTCAGAAGGCTC |  |  |  |
|  |  | GM-CSF_P | CACTCGCCAACCCAGCCCTG |  |  |  |
| TGF-β | Transforming growth factor beta | TGF-β_F | CCTGCTGAGGCTCAAGTTAAAAGT | 82 | 102 |  |
|  |  | TGF-β_R | CTGAGGTAGCGCCAGGAATT |  |  |  |
|  |  | TGF-β_P | TGCTATATTTCTGGTACAGCTCCACGTGCTG |  |  |  |
| IL-1α | Interleukin-1 alpha | IL-1α_F | CGAGATATGTCAGGTCCATA | 75 | 99 | Primer 3 |
|  |  | IL-1α_R | AACCATGTCAAATTTCACTG |  |  |  |
|  |  | IL-1α_P | CATGACGGCTGCTACATTAAAT |  |  |  |
| IL-6 | Interleukine-6 | IL-6_F | TCACAAGCGCCTTCAGTCCA | 121 | 103 |  |
|  |  | IL-6_R | AGTCTGCTTGGGGTGGTGTC |  |  |  |
|  |  | IL-6_P | TGGGGCTGCTCCTGGTGATGACT |  |  |  |
| IL-18 | Interleukine-18 | IL-18_F | TGTAGCTGAAAATGGCGAAGAC | 170 | 103 |  |
|  |  | IL-18_R | TGGTCTGGGGTGCATTATCTGA |  |  |  |
|  |  | IL-18_P | ACGACCAAGTTCTCTTCATTAGCCAGGGA |  |  |  |
| IL-12β | Interleukine-12β | IL-12β_F | TGGAGTGTCAGGAGGGCAGT | 150 | 101 |  |
|  |  | IL-12β_R | GGTTCTTGGGTGGGTCTGGT |  |  |  |
|  |  | IL-12β_P | GGAGAGCCTGCCCATTGAGGTCGT |  |  |  |
| CCL5 | C-C chemokine ligand 5 (RANTES) | CCL5_F | TGCTTTGCCTACCTCTCCCG | 138 | 105 |  |
|  |  | CCL5_R | CTTCTTCTCTGGGTTGGCGC |  |  |  |
|  |  | CCL5_P | TGCCCCGCAACCACGTCCAGGAAT |  |  |  |
| CCR5 | chemokine receptor 5 | CCR5_F | CCAACAAGTGTGGCCAGCAG | 187 | 112 |  |
|  |  | CCR5_R | GCCTCCACTGATGCTCCACT |  |  |  |
|  |  | CCR5_P | TCCTTGATCAGAGACGCAGACGGGACA |  |  |  |
| CXCL1 | C-X-C chemokine ligand 1 | CXCL1_F | ATGCAGAGCGTGAAGGTGAC | 154 | 90 |  |
|  |  | CXCL1_R | AGTTGGAGCTGGCCTGGTTT |  |  |  |
|  |  | CXCL1_P | CCCCGCCGCCCCCATGGTTAAGAAA |  |  |  |
| CXCL11 | C-X-C chemokine ligand 11 | CXCL11_F | AGTCCTGGCTGTGATATTATGTG | 110 | 104 | Primer Questool |
|  |  | CXCL11_R | TATCTGCCACTTTCACTGCTT |  |  |  |
|  |  | CXCL11_P | ACTCCAGGGCCTATGCAAAGACAC |  |  |  |
| CASP1 | Caspase-1 | CASP1_F | CTGCTCTTCGACACCAGATAAT | 106 | 90 |  |
|  |  | CASP1_R | GTCACAGGACCAGGCATATT |  |  |  |
|  |  | CASP1_P | CCTGGAGACATCCCATATTCGGCT |  |  |  |
| OAS1 | 2'-5'-oligoadenylate synthetase 1 | OAS1_F | AGTTCTCCCCCTGCTTCACGGA | 147 | 109 | Primer 3 |
|  |  | OAS1_R | CCAGGGCATATTGTGGGGGCAA |  |  |  |
|  |  | OAS1_P | GCCTCATCCGCCTGGTGAAGCACTGGT |  |  |  |
| MX1 | Myxovirus resistance gene-1 | Mx1_F | ACTTGGTGGTGGTCCCTGCT | 133 | 100 |  |
|  |  | Mx1_R | TCCGTGCCTTTGTCCACCAGA |  |  |  |
|  |  | Mx1_P | CGCTGCGCATGGCTCAGGATGTGGA |  |  |  |
| MDA5 | Melanoma Differentiation-Associated protein 5 (Interferon-induced with helicase C domain 1) | MDA5_F | TCGGCTGGGCTAGGATGTTCGT | 81 | 107 |  |
|  |  | MDA5_R | TGAGGTCTGGGTTCACGTAGCG |  |  |  |
|  |  | MDA5_P | TCCGGCAAGCAGGCAACCCCTTAGC |  |  |  |
| ISG15 | Interferon-stimulating gene 15 | ISG15_F | CGCCCAGAAGATCAATGTGCCT | 159 | 89 |  |
|  |  | ISG15_R | AGGATGTTCAGGGTGGCGCT |  |  |  |
|  |  | ISG15_P | CCCCTTGTCCACCAGGGCCTGAAAGCT |  |  |  |
| TLR3 | Toll-like receptor 3 | TLR3_F | CACCCTCTTCCGAACCTGGT | 168 | 99 |  |
|  |  | TLR3_R | CAGAAACAGAACAGGGCCGC |  |  |  |
|  |  | TLR3_P | TGCAGCATAACAACTTAGCTCGGCTCTGG |  |  |  |
| TLR7 | Toll-like receptor 7 | TLR7_F | GCCAATGCCACCAACCTCAC | 128 | 89 |  |
|  |  | TLR7_R | GGCCCCAGTCGAACAGGTAT |  |  |  |
|  |  | TLR7_P | ATCTCTCCAGCCTCCTTCCACCGGC |  |  |  |


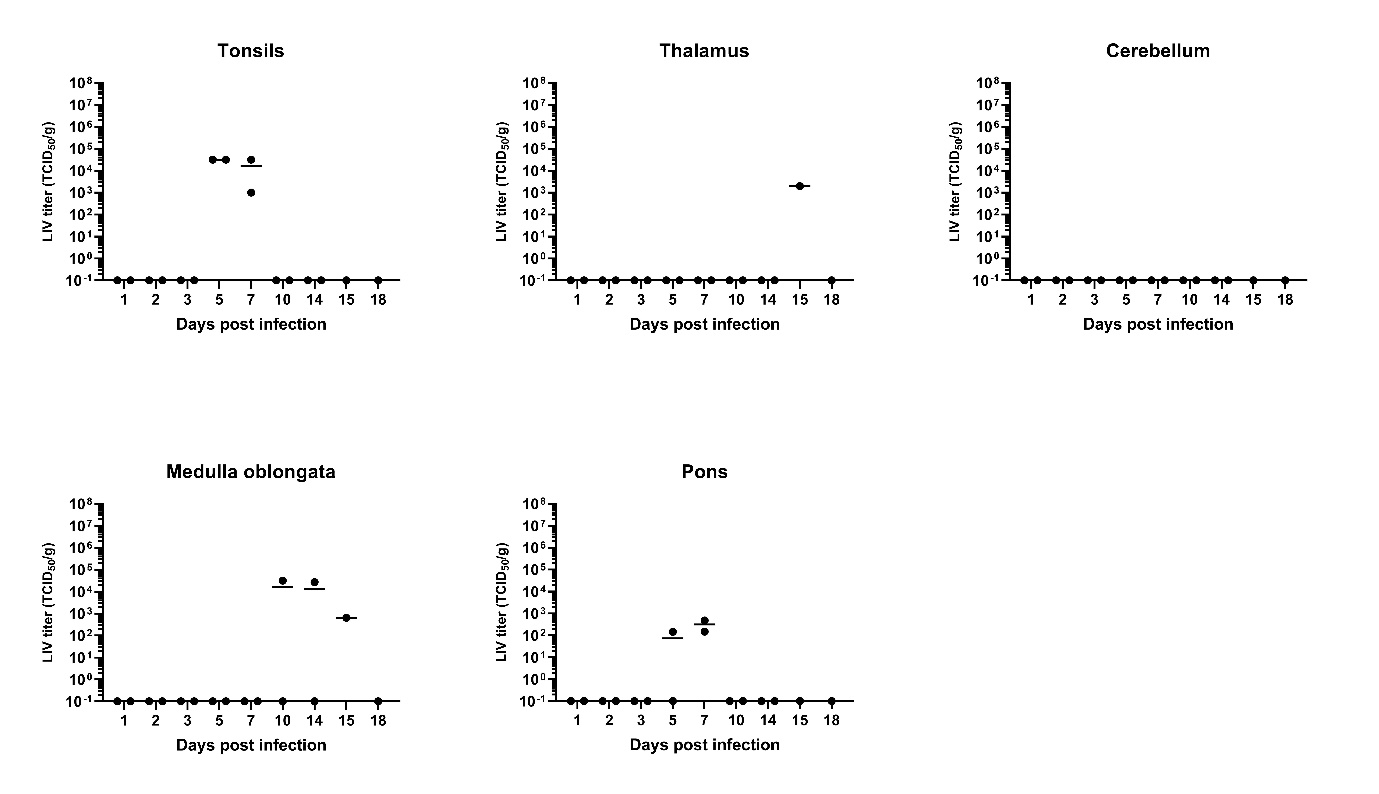


**Figure S 1** Amounts of infectious LIV in the soft palate tonsil, thalamus, cerebellum, medulla oblongata and pons upon intradermal inoculation of 8-months-old sheep with 10^5^ TCID_50_/animal of LIV LI/31 strain, determined by virus titration and expressed as TCID_50_/g.


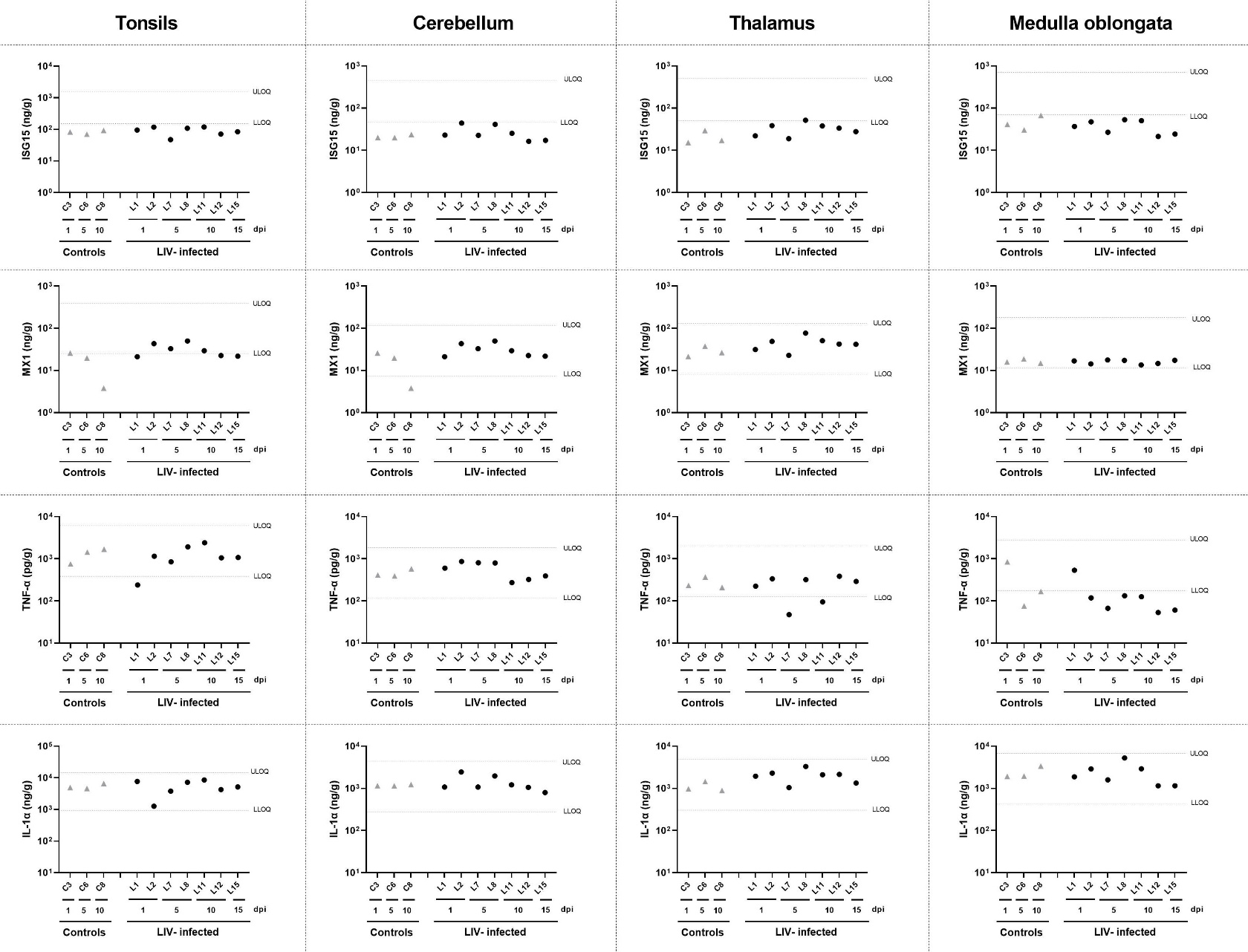


**Figure S 2** ELISA analysis of ISG15, MX1, TNF-α and IL-1α levels in the tonsils, cerebellum, thalamus and medulla oblongata of 8-month-old sheep inoculated intradermally with LIV LI/31 strain at a dose of 10^5^ TCID_50_/sheep. Protein concentrations were normalized to tissue weight and were expressed as pg/g or ng/g depending on the ELISA. The upper limit of quantification (ULOQ) and lower limit of quantification (LLOQ) were determined using the upper and lower limits of the standard curve range.


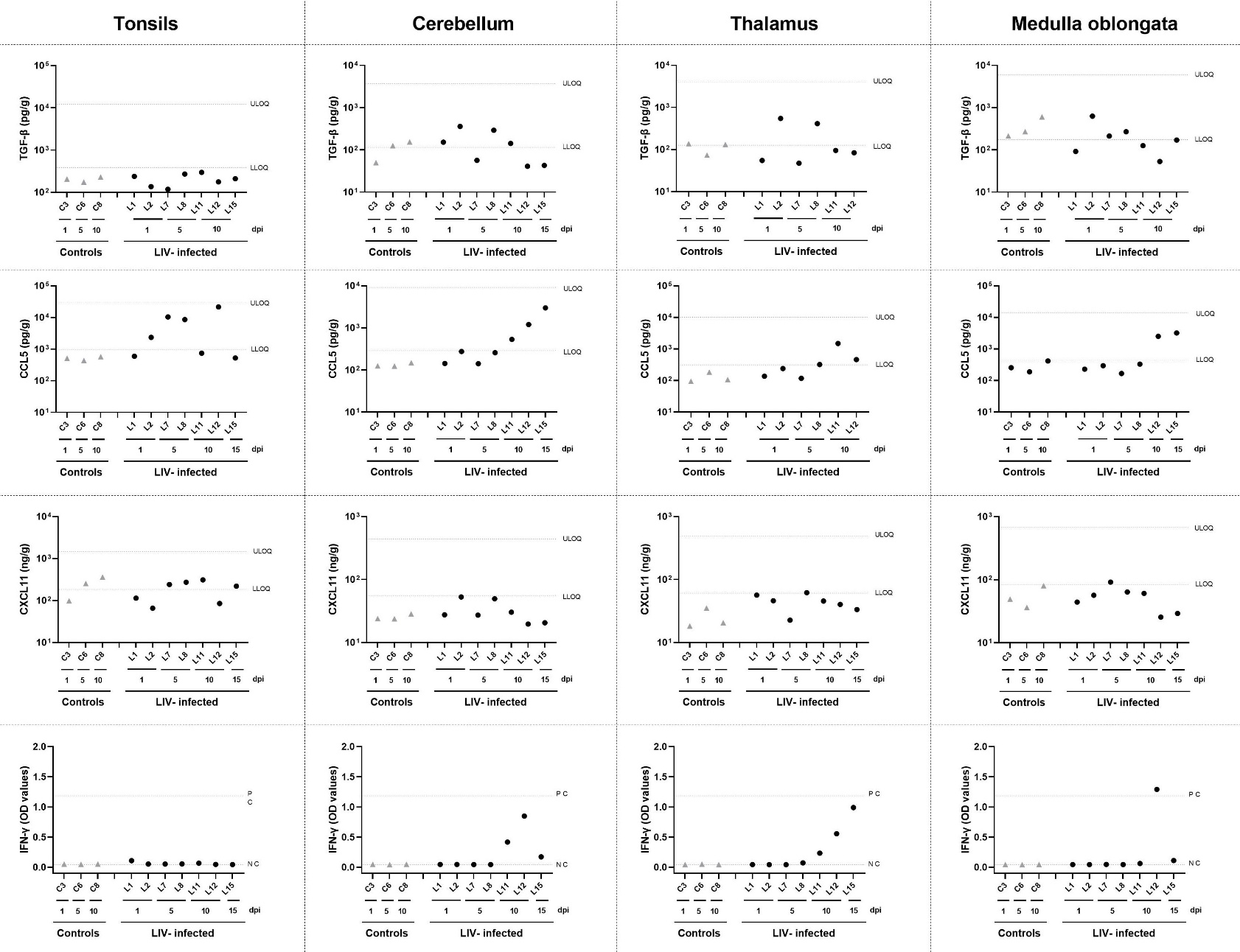
**Figure S 3** ELISA analysis of TGF-β, CCL5, CXCL11 and IFN-γ levels in the tonsils, cerebellum, thalamus and medulla oblongata of 8-month-old sheep inoculated intradermally with LIV LI/31 strain at a dose of 10^5^ TCID_50_/sheep. Protein concentrations of TGF-β, CCL5 and CXCL11 were normalized to tissue weight and were expressed as pg/g or ng/g depending on the ELISA. The upper limit of quantification (ULOQ) and lower limit of quantification (LLOQ) were determined using the upper and lower limits of the standard curve range. For IFN-γ, sample reactivity was determined by comparing optical density (OD) values of samples to those of the positive (PC) and negative controls (NC).


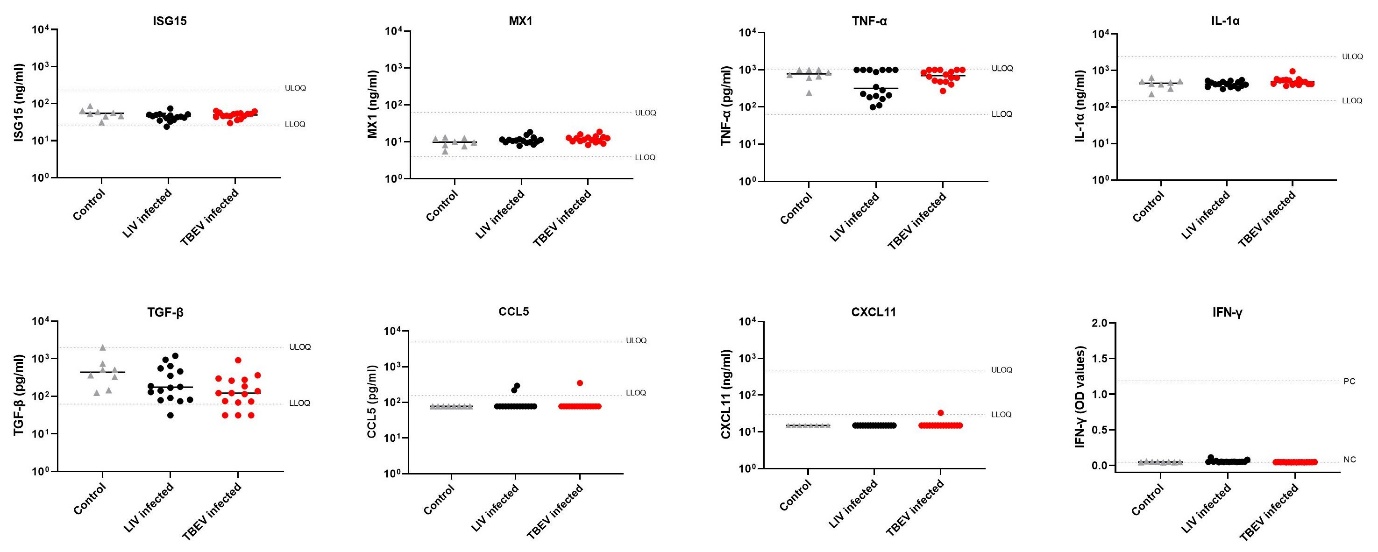


**Figure S 4** ELISA analysis of ISG15, MX1, TNF-α, IL-1α, TGF-β, CCL5, CXCL11 and IFN-γ levels in the serum of 8-month-old sheep inoculated intradermally with LIV LI/31 strain or TBEV Neudoerfl strain at a dose of 10^5^ TCID_50_/sheep. Protein concentrations were expressed as pg/ml or ng/ml depending on the ELISA. The upper limit of quantification (ULOQ) and lower limit of quantification (LLOQ) were determined using the upper and lower limits of the standard curve range. For IFN-γ, sample reactivity was determined by comparing optical density (OD) values of samples to those of the positive (PC) and negative controls (NC).
